# Putative Regulatory Factors Associated with Intramuscular Fat Content

**DOI:** 10.1371/journal.pone.0128350

**Published:** 2015-06-04

**Authors:** Aline S. M. Cesar, Luciana C. A. Regitano, James E. Koltes, Eric R. Fritz-Waters, Dante P. D. Lanna, Gustavo Gasparin, Gerson B. Mourão, Priscila S. N. Oliveira, James M. Reecy, Luiz L. Coutinho

**Affiliations:** 1 Department of Animal Science, University of São Paulo, Piracicaba, SP, 13418–900, Brazil; 2 Embrapa Southeast-Cattle Research Center, São Carlos, SP, 13560–970, Brazil; 3 Department of Animal Science, Iowa State University, Ames, IA, 50011, United States of America; 4 Department of Genetics and Evolution, Federal University of São Carlos, São Carlos, SP, 13565–905, Brazil; University of Bologna, ITALY

## Abstract

Intramuscular fat (IMF) content is related to insulin resistance, which is an important prediction factor for disorders, such as cardiovascular disease, obesity and type 2 diabetes in human. At the same time, it is an economically important trait, which influences the sensorial and nutritional value of meat. The deposition of IMF is influenced by many factors such as sex, age, nutrition, and genetics. In this study Nellore steers (*Bos taurus indicus* subspecies) were used to better understand the molecular mechanisms involved in IMF content. This was accomplished by identifying differentially expressed genes (DEG), biological pathways and putative regulatory factors. Animals included in this study had extreme genomic estimated breeding value (GEBV) for IMF. RNA-seq analysis, gene set enrichment analysis (GSEA) and co-expression network methods, such as partial correlation coefficient with information theory (PCIT), regulatory impact factor (RIF) and phenotypic impact factor (PIF) were utilized to better understand intramuscular adipogenesis. A total of 16,101 genes were analyzed in both groups (high (H) and low (L) GEBV) and 77 DEG (FDR 10%) were identified between the two groups. Pathway Studio software identified 13 significantly over-represented pathways, functional classes and small molecule signaling pathways within the DEG list. PCIT analyses identified genes with a difference in the number of gene-gene correlations between H and L group and detected putative regulatory factors involved in IMF content. Candidate genes identified by PCIT include: *ANKRD26*, *HOXC5* and *PPAPDC2*. RIF and PIF analyses identified several candidate genes: *GLI2* and *IGF2* (RIF1), *MPC1* and *UBL5* (RIF2) and a host of small RNAs, including miR-1281 (PIF). These findings contribute to a better understanding of the molecular mechanisms that underlie fat content and energy balance in muscle and provide important information for the production of healthier beef for human consumption.

## Introduction

Intramuscular fat (IMF, also known as marbling) represents the amount of fat accumulated between muscle fibers or within muscle cells, which is the sum of phospholipids (present in cell membranes), and triglycerides (lipid droplets). Understanding the biological and functional mechanisms that regulate IMF content is an interesting issue in meat science and human medicine. Intramuscular fat content is a polygenic trait regulated by many genes involved directly, or indirectly in adipogenesis and fat metabolism [1]. The deposition of IMF is influenced by many factors such as sex, age, breed, nutrition, and genetics [2].

High intramuscular fat content (marbling) has been associated with tenderness, juiciness and consumer satisfaction [3]. At the same time, red meat consumption or more specifically saturated fat consumption has been associated with human diseases, such as cardiovascular disease, obesity and colon cancer [4]. These associations have created a demand for beef with both low fat and high quality by consumers. In addition, consumers in some countries have different preferences of IMF amount. For example, consumers in Asia and North America desire beef with high IMF, while Europeans prefer lean beef, with low IMF [5].

RNA-Seq has been used to provide insight into biological and molecular mechanisms of complex traits and diseases. Recent RNA-Seq studies of pigs with extreme IMF phenotypes have defined differentially expressed genes (DEG) and potential gene networks important in lipid and fatty acid metabolism in the liver [6]. In beef cattle, DEG were identified in subcutaneous fat from *Bos taurus* crossed steers, which revealed that expression pattern depends on the genetic background [7]. Two studies have investigated DEG due to IMF deposition in *Bos taurus*, *Bos indicus* and their crosses [8, 9], however the biological and functional mechanisms involved with IMF amount are still unclear. From the published literature, lipid metabolism is considerably different between nonruminants and ruminants, but gene expression studies in ruminant are relatively scarce.

Understanding the biological mechanisms involved with complex traits requires analysis of genetic networks, as well as determination of relationships between genes and networks. A novel algorithm for the partial correlation coefficient with information theory (PCIT) mathematical method was developed to determine the relationships between all genes and networks [10]. Previously, the PCIT algorithm approach identified causal regulatory changes in myostatin gene expression in beef cattle [11] by co-expression network analysis and the regulatory and phenotypic impact factor methods (RIF and PIF). These co-expression methods provide powerful incite into the changes that occur in the network makeup and wiring between different treatment groups. These methods allow subtle changes in networks to be detected even when many genes in a pathway are not differentially expressed.

The objective of this study was to identify differentially expressed genes, pathways and putative regulators associated with IMF variation in *Longissimus dorsi* muscle of extreme IMF GEBV Nellore steers to better understanding of IMF metabolism.

## Materials and Methods

### Ethics statement

All experimental procedures involving steers were approved by the Institutional Animal Care and Use Committee Guidelines from Brazilian Agricultural Research Corporation—EMBRAPA and sanctioned by the president Dr. Rui Machado.

### Animals, samples and phenotypes

Three hundred and ten Nellore steers from the Brazilian Agricultural Research Corporation (EMBRAPA/Brazil) experimental breeding herd, raised between 2009 and 2011 were included in this study, as described by Cesar and collaborators [12]. These steers were sired by 34 unrelated sires, and were selected to represent the main genealogies used in Brazil according to the National Summary of Nellore produced by the Brazilian Association of Zebu Breeders (ABCZ) and National Research Center for Beef Cattle. Animals were raised in feedlots under identical nutrition and handling conditions until slaughter at an average age of 25 months. Samples from LD muscle located between the 12th and 13th ribs were collected in two moments: at slaughter for RNA sequencing analysis, and 24 hours after slaughter for the intramuscular fat (IMF) content measurement.

Approximately 100 g samples of beef collected for IMF content analysis were lyophilized and ground to a fine powder. Five g of this ground, lyophilized tissue was used to obtain IMF. The Ankom XT20 lipids equipment was used to determine lipid content according to the procedure of AOCS (Official Procedure Am 5–04) for IMF extraction [13]. Restricted maximum likelihood analysis was performed to estimate variance components, heritability and Genomic Best Linear Unbiased Prediction (GBLUP) using ASREML software [14] on 310 total animals. The SNP markers information was obtained as described by Cesar and collaborators (2014) using BovineHD 770 k BeadChip (Infinium BeadChip, Illumina, San Diego, CA). SAS PROC MIXED was used to test independent sources for significance. Fixed effects included contemporary group classes (animals with the same origin, birth year and slaughter date) and hot carcass weight as a covariate. Animal and residuals were fitted as random effects [12]. The animal model used in this analysis was, ***y*** = ***Xb*** + ***Zu*** + ***e***, where **y** is the vector of observations, which represented the trait of interest (dependent variable), **X** and **Z** are the design or incidence matrices for the vectors of fixed and random effects in **b** and **u,** respectively, and **e** was the vector of random residuals. The expected variance of vector **u** is Var(a) = I σ^2^_m_; where σ^2^_m_ is the variance explained by markers, and **I** is the identity matrix. The variance of vector **u** was **G** σ^2^_m_ for the genomic analyses where **G** is the genomic relationship matrix derived from SNP markers using allele frequencies as suggested by VanRaden [15], with σ^2^_m_ being the marker-based additive genetic variance. A group of 14 animals were selected based on their extreme genomic estimated breeding values (GEBVs) for IMF (seven high and seven low). To verify the difference in IMF level between the high and low group a Student's t-test was performed using R package. The genomic estimated breeding values (GEBVs) for other muscle characteristics such as ribeye area and backfat thickness were also calculated to ascertain these animal were not extreme for another characteristic.

### RNA extraction, quality analysis, library preparation and sequencing

Total RNA was extracted from 100 mg of frozen LD muscle that was collected at slaughter using the TRIzol reagent (Life Technologies, Carlsbad, CA). RNA integrity was verified by Bioanalyzer 2100 (Agilent, Santa Clara, CA, USA). Only samples with RIN > 8 were used. A total of 2μg of total RNA from each sample was used for library preparation according to the protocol described in the TruSeq RNA Sample Preparation kit v2 guide (Illumina, San Diego, CA). Libraries average size was estimated using the Agilent Bioanalyzer 2100 (Agilent, Santa Clara, CA, USA) and quantified using quantitative PCR with the KAPA Library Quantification kit (KAPA Biosystems, Foster City, CA, USA). Quantified, samples were diluted and pooled (three pools of six samples each). Three lanes of a sequencing flowcell, using the TruSeq PE Cluster kit v3-cBot-HS kit (Illumina, San Diego, CA, USA), were clusterized and sequenced using HiScanSQ equipment (Illumina, San Diego, CA, USA) with a TruSeq SBS Kit v3-HS (200 cycles), according to manufacturer instructions. The sequencing analyses were performed at the Genomics Center at ESALQ, Piracicaba, São Paulo, Brazil.

### Quality control and read alignment

Sequencing adaptors and low-complexity reads were removed in an initial data-filtering step. Quality control and reads statistics were estimated with FASTQC version 0.10.1 software [http://www.bioinformatics.bbsrc.ac.uk/projects/fastqc/]. Tophat v. 1.2.0 software [16] was used to map reads to the UMD3.1 *Bos taurus* reference assembly available at Ensembl [http://www.ensembl.org/Bos_taurus/Info/Index/]. A reference-guided assembly was performed using Cufflinks version 2.0.2, with a minimum alignment count per locus of 10 per transcript [17], to identify novel transcripts. A combination of novel transcripts identified by Cufflinks and those from the reference GTF file at Ensembl were used as the reference for read quantification for each transcript. The abundance (read counts) of mRNAs for all annotated genes, was calculated using HTSeq version 0.5.4 software [http://www-huber.embl.de/users/anders/HTSeq/] [18]. Only sequence reads that uniquely mapped to known chromosomes (excluding reads mapped to unassigned contigs) were used in this analysis.

### Identification of differential expressed genes and pathway analysis

Differentially expressed genes were identified using the DESeq software according to the protocol proposed by Anders and collaborators [18], which uses read counts that fall into annotated genes and perform statistical analysis based on the table of counts to discover quantitative changes in expression levels between experimental groups. Prior to statistical analysis, the read count data was filtered as follows: i) transcripts with zero counts were removed (unexpressed); ii) transcripts with less than 1 read per sample on average were removed (very lowly expressed); iii) transcripts that were not present in at least three samples were removed (rarely expressed). After filtering, a total of 16,101 transcripts were analyzed for differential expression using the “nbinomTest” function of DESeq to fit transcript expression level as a negative binomial distribution. Exploratory diagnostic plots were generated to check the dispersion estimates. Benjamini-Hochberg [19] methodology was used to control the false discovery rate (FDR) at 10%. Transcript annotations were retrieved with a perl script to query the Ensembl database using the Ensembl Perl Application Program Interface (API). Transcripts that lacked annotation information were annotated using the Genome-to-seq and GOanna for GO annotations based on sequence homology by Basic Local Alignment Search Tool (BLAST) at AgBase [20]. A two-tiered approach was taken to detect pathway level changes in gene ontology in the extreme IMF samples RNAseq profiles. First, enrichment analysis of curated gene ontology terms was completed with the Database for Annotation, Visualization and Integrated Discovery (DAVID) v6.7 tool [21] using the list of genes that presented FDR < 10%. Second, a literature based pathway enrichment analysis was performed using Pathway Studio [22] from a list of genes that presented FDR < 20%.

### PCIT and differential hubbing network analysis

Expression values were normalized as the number of fragments per kilobase of exon per million reads (FPKM) as reported in Cufflinks output [17] for co-expression analysis. A modified version of the PCIT algorithm [23] was used to identify differential hubbing (DH) for all transcripts [11]. A shell script pipeline was developed to summarize all DH results. The gene list used for PCIT included all transcripts detected in our study, but only those with a direct and partial correlation ≥ 0.90 were used for the DH analysis. The DH was computed by the difference of significant connections of a transcript between the low and high IMF group. The top ten most positively and negatively DH transcripts were identified for further investigation.

### Regulatory Impact Factor (RIF) network co-expression analysis and phenotypic impact factor (PIF) scores

The regulatory impact factor (RIF) and phenotypic impact factor (PIF) scores were calculated as described [24] to predict which transcripts were potential regulators of gene expression differences between the high and low IMF groups. The RIF calculations presented here were modified from the original method and the complete list of expressed transcripts were tested as potential regulators and only transcripts with a significant partial correlation ≥ 0.90 from PCIT were included in the RIF and PIF score estimates.

The PIF values represent a way to rank genes based on the magnitude of the expression of a gene and the difference in the expression of that gene between two treatments. A high PIF value would indicate that a given gene would likely be closely related to changes in phenotype. The RIF1 value allows genes to be ranked as potential regulators of networks based largely on changes in correlation between two treatment levels (i.e. differential wiring). The RIF2 value allows genes to be ranked as potential regulators of networks with more of an emphasis on how expression changes of a potential regulator may predict the abundance of genes DE due to treatment differences [24]. Thus, RIF2 ranks genes as potential biomarkers tracking key differences in gene expression related to treatment differences.

## Results

### Phenotypic groups, mapping and annotation

A summary of the IMF phenotypic data expressed as a percentage, GEBVs, and the total number of reads mapped against the *Bos taurus* UMD3.1 reference genome assembly following quality control are shown in Table 1. The genetic variance, residual variance and heritability for IMF obtained from this population were 0.196, 0.490 and 0.29±0.16, respectively. The GEBV for IMF values for all 310 animals were ranked and seven animals with high GEBV for IMF (H) and seven with low (L) were selected for RNA-Seq analysis. This strategy, to select the animals with extreme GEBV was performed for two main reasons: (1) the correlation between the raw IMF values (percentage of IMF) and GEBV was high (r = 0.76) (S1 Fig) and (2) the GBLUP procedure used genomic information from all relatives and can account for environmental effects [25]. The T-test showed that the IMF averages for two groups were statistically different (p-value = 2.016e-09). The cattle used in this study were not extreme individuals for either subcutaneous fat thickness or longissimus muscle area (S1 Table). These results indicate that the samples selected for IMF were not divergent for other characteristics of muscle measured in this population.

**Table 1:** Intramuscular fat percentage (IMF), genomic estimated breeding values (GEBV) and mapped reads for all animals within group (Low and High) based on IMF GEBV.

Animal	IMF (%)	GEBV	Mapped reads (M)^1^
Low_1_ ^2^	1.70	-0.31	23.09
Low_2_	1.94	-0.29	11.13
Low_3_	1.86	-0.24	25.11
Low_4_	1.60	-0.59	13.32
Low_5_	1.32	-0.77	14.34
Low_6_	1.58	-0.50	14.58
Low_7_	1.62	-0.57	17.85
High_1_^3^	4.42	0.44	22.65
High_2_	4.35	0.57	17.53
High_3_	4.38	0.71	17.58
High_4_	5.27	0.85	16.31
High_5_	5.02	0.47	15.73
High_6_	4.74	0.81	15.13
High_7_	4.35	0.61	13.76
Mean Low	1.56 ± 0.20^2^	-0.45	17.04
Mean High	4.65 ± 0.37^2^	0.65	19.39

^1^M million reads

^2^ Standard deviation (SD)

A total of 364.18 million (M) 100 bp paired-end reads were obtained from three lanes of an Illumina HiScanSQ. The average number of total reads per sample was 26 M, with an estimated sequencing depth of 40X coverage (i.e. the expected mean coverage at all transcripts). The mean number of total mapped reads estimated by Tophat [16] was 17 M, for an average of 65.38% paired reads mapped. All transcripts were annotated using the Ensembl Perl API or AgBase tools [20], genome2seq and GOanna. A total of 16,101 genes were analyzed after filtering out lowly expressed genes. Correlations among mean gene expression levels between both the H and L groups was high (r = 0.99).

### Differential expression analysis and differentially expressed genes

The DESeq R package was used to identify DEG. This statistical package uses a parametric method, which relies on assumptions regarding the distribution of sampled data [18]. The inference relies on estimation of the typical relationship between the mean of gene expression levels and their data’s dispersion (square of the coefficient of biological variation). Dispersion plot of the 16,101 expressed genes identified in both groups (H and L) is presented in S2 Fig. Using this parametrical approach and extending Fisher’s exact test to the data following a negative binomial distribution, 77 DEG (false discovery rate (FDR 10%) were detected (S3 Fig and S2 Table) between the H and L IMF groups. The histogram of p values demonstrates the presence of DEG (S4 Fig). Of the 77 DEG, 41 genes were up-regulated and 36 down-regulated in the L group (S2 Table). The expression level, fold change, p-value and annotation of all 16,101 genes identified are showed in the S3 Table.

### Functional enrichment and pathways

**Fig 1:**
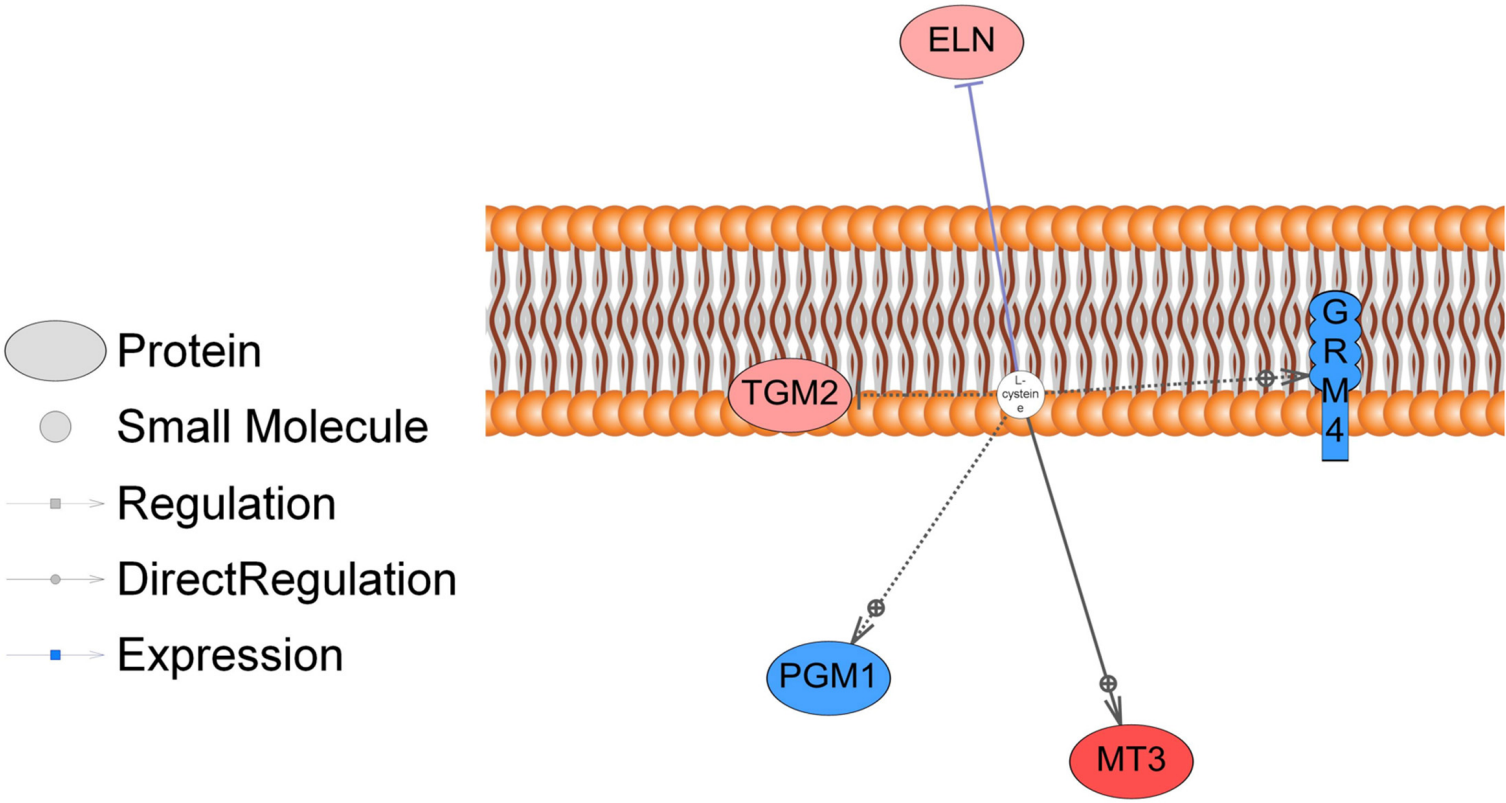
L-cysteine pathway genes identified as differentially expressed between the high and low groups for IMF GEBV are shown here (FDR ≤ 0.10, adjusted for multiple testing using Benjamini-Hochberg method). The genes shown in red had higher expression in the low IMF group and those in blue had higher expression in animals from the High IMF group.

A gene-annotation enrichment analysis was performed using the DAVID software [21] and knowledgebase to capture enriched biological terms from the DEG list (FDR < 10%). This analysis identified (Table 2) two significant KEGG pathways based on Benjamini-Hochberg (BH) methodology [19]: focal adhesion (BH-adj < 0.02) and Extracellular matrix (ECM)-receptor interaction (BH-adj < 0.056). A gene set enrichment analysis (GSEA) was performed to identify over-represented pathways in DEG (FDR < 20%) using Pathway Studio. The GSEA detected 13 pathways (FDR < 10%) that contained 27 DEG (Table 3). These DEG are associated with multiple pathways, which could indicate an inter-relation between the pathways. An over-representation of genes were identified in the following functional classes by Pathway Studio: L-cysteine, Zn^2+^, H_2_O_2_, Mg^2+^, ATP, retinoic acid, NO, ROS, oxidized LDL complex, inflammatory cytokines, and protein tyrosin-kinase. Pathway Studio also identified genes associated with IL-1ß and NF-kB pathways. Fig 1 shows the genes associated with the L-cysteine small molecule, and the genes associated with the retinoic acid and inflammatory cytokine functional classes are shown in S5 and S6 Figs, respectively, where genes colored in red are overexpressed in animals of Low group and those in blue are overexpressed in H group.

**Table 2:** Functional enrichment and significant category (BH-adj < 0.10) are shown from DEG (FDR < 0.10) comparing high and low IMF GEBV animals.

Category	Term	Count^1^	%^2^	P-Value	Benjamini
KEGG_PATHWAY	Focal adhesion	6	8.0	4.9E-4	2.0E-2
GOTERM_BP_FAT	cellular protein catabolic process	7	9.3	2.3E-4	4.6E-2
GOTERM_BP_FAT	protein catabolic process	7	9.3	3.5E-4	4.7E-2
GOTERM_BP_FAT	cellular macromolecule catabolic process	7	9.3	5.4E-4	5.3E-2
KEGG_PATHWAY	ECM-receptor interaction	4	5.3	2.7E-3	5.6E-2
SP_PIR_KEYWORDS	basement membrane	3	4.0	6.9E-4	6.0E-2
GOTERM_BP_FAT	macromolecule catabolic process	7	9.3	1.0E-3	7.9E-2
GOTERM_BP_FAT	modification-dep. protein catabolic process	6	8.0	1.2E-3	8.0E-2
GOTERM_BP_FAT	modification-dep. macromolecule catabolic process	6	8.0	1.2E-3	8.0E-2
GOTERM_BP_FAT	proteolysis involved in cell. protein catabol. process	7	9.3	2.3E-4	8.8E-2
GOTERM_BP_FAT	muscle tissue development	4	5.3	1.9E-3	9.1E-2
GOTERM_BP_FAT	striated muscle tissue development	4	5.3	1.6E-3	9.1E-2

^1^ Number of differentially expressed genes (DEG) involved in the term

^2^ Percentage of DEG involved in the term (involved DEG/total DEG)

**Table 3:** Gene Set Enrichment Analysis, significant functional classes and pathways (p < 0.10) are shown from DEG (FDR < 0.20) comparing high and low IMF GEBV animals.

Name	Type	Total number of Neighbors	Number of Measured Neighbors	Gene Set Seed	Neighbors	padj^1^
Neighbors of L-cysteine	Pathway	366	5	L-cysteine	*MT3*, *GRM4*, *PGM1*, *TGM2*, *ELN*	0.011
Neighbors of Zn2+	Pathway	905	6	Zn2+	*MT3*, *ADAMTS12*, *PGM1*, *TGM2*, *SLC2A4*, *STAT5A*	0.021
Neighbors of oxidized LDL	Pathway	424	5	oxidized LDL	*PTAFR*, *COL4A2*, *TGM2*, *SERPINE2*, *SLC2A4*	0.023
Neighbors of inflammatory cytokine	Pathway	774	9	inflammatory cytokine	*MT3*, *ADAMTS12*, *FBXO32*, *CRYAB*, *TGM2*, *SERPINE2*, *SLC2A4*, *ELN*, *STAT5A*	0.025
Neighbors of H2O2	Pathway	1142	8	H2O2	*MT3*, *FBXO32*, *CRYAB*, *HTATIP2*, *TGM2*, *SLC2A4*, *TGFB1I1*, *ELN*	0.030
Neighbors of Mg2+	Pathway	562	5	Mg2+	*ADAMTS12*, *PGM1*, *TGM2*, *REM1*, *ELN*	0.033
Neighbors of ATP	Pathway	1167	9	ATP	*GRM4*, *PGM1*, *FBXO32*, *CRYAB*, *SLC4A4*, *TGM2*, *SLC2A4*, *GYPC*, *NRP1*	0.061
Neighbors of IL1B	Pathway	1191	12	IL1B	*MT3*, *CSRP3*, *RGS16*, *FBXO32*, *CRYAB*, *TGM2*, *SERPINE2*, *SLC2A4*, *SPARC*, *ELN*, *STAT5A*, *NRP1*	0.063
Neighbors of retinoic acid	Pathway	1605	12	retinoic acid	*SLC7A4*, *CSRP3*, *PTAFR*, *RGS16*, *CPM*, *TGM2*, *SLC2A4*, *FLNA*, *SPARC*, *ELN*, *STAT5A*, *ROCK2*	0.074
Neighbors of NF-kB	Pathway	1125	9	NF-kB	*MT3*, *PTAFR*, *RGS16*, *FBXO32*, *TGM2*, *SLC2A4*, *ELN*, *STAT5A*, *FSCN1*	0.085
Neighbors of protein tyrosine kinase	Pathway	823	7	protein tyrosine kinase	*PGM1*, *PTAFR*, *RGS16*, *SLC4A4*, *SLC2A4*, *STAT5A*, *BCR*	0.092
Neighbors of NO	Pathway	862	8	NO	*CSRP3*, *PTAFR*, *FBXO32*, *TGM2*, *SLC2A4*, *SPARC*, *ELN*, *STAT5A*	0.097
Neighbors of ROS	Pathway	787	8	ROS	*MT3*, *PTAFR*, *SLC4A4*, *TGM2*, *SLC2A4*, *TGFB1I1*, *ELN*, *STAT5A*	0.098

^**1**^ padj—p value adjusted for multiple testing with the Benjamini-Hochberg procedure, which controls false discovery rate (FDR).

### PCIT and differential hubbing analysis

The PCIT algorithm was used to identify differential hubbing (DH) between the H and L IMF groups. Differential hubbing (DH) or differential connectivity (DC) is the difference in the number of significant partial correlations a gene has between two different states (i.e. compared between high and low groups), as computed by the PCIT algorithm. In other words, DH is the change in the number of significant connections between two states. The significance of a correlation is determined using an information theory approach in the case of the PCIT method.

**Fig 2:**
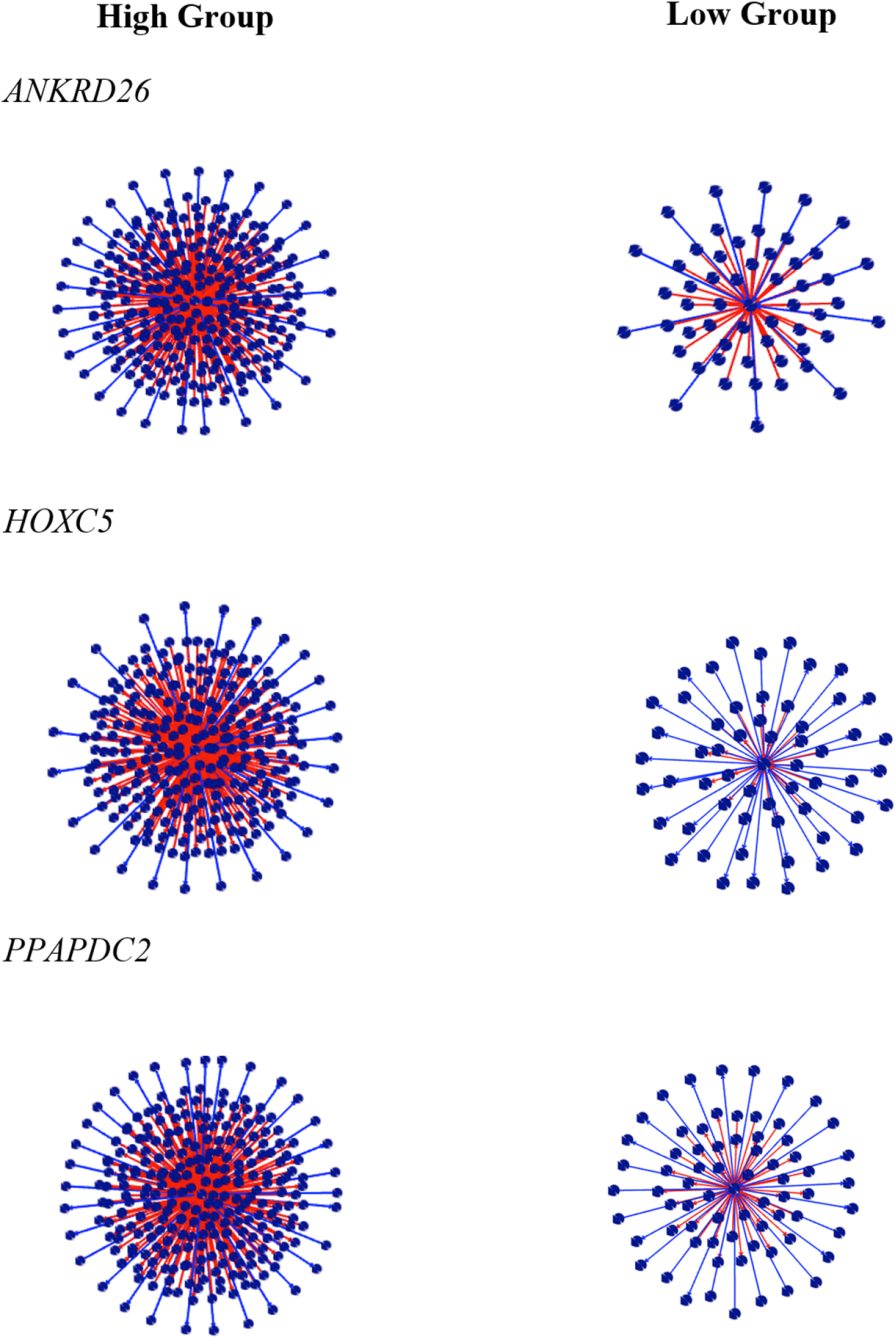
Positive differential hubbing (DH) between the high and low groups for IMF GEBV. The center spot represents the gene with high value of DH, the red edges represent the positive DH and blue edges represent the negative DH. Other spots represent the connections.

**Fig 3:**
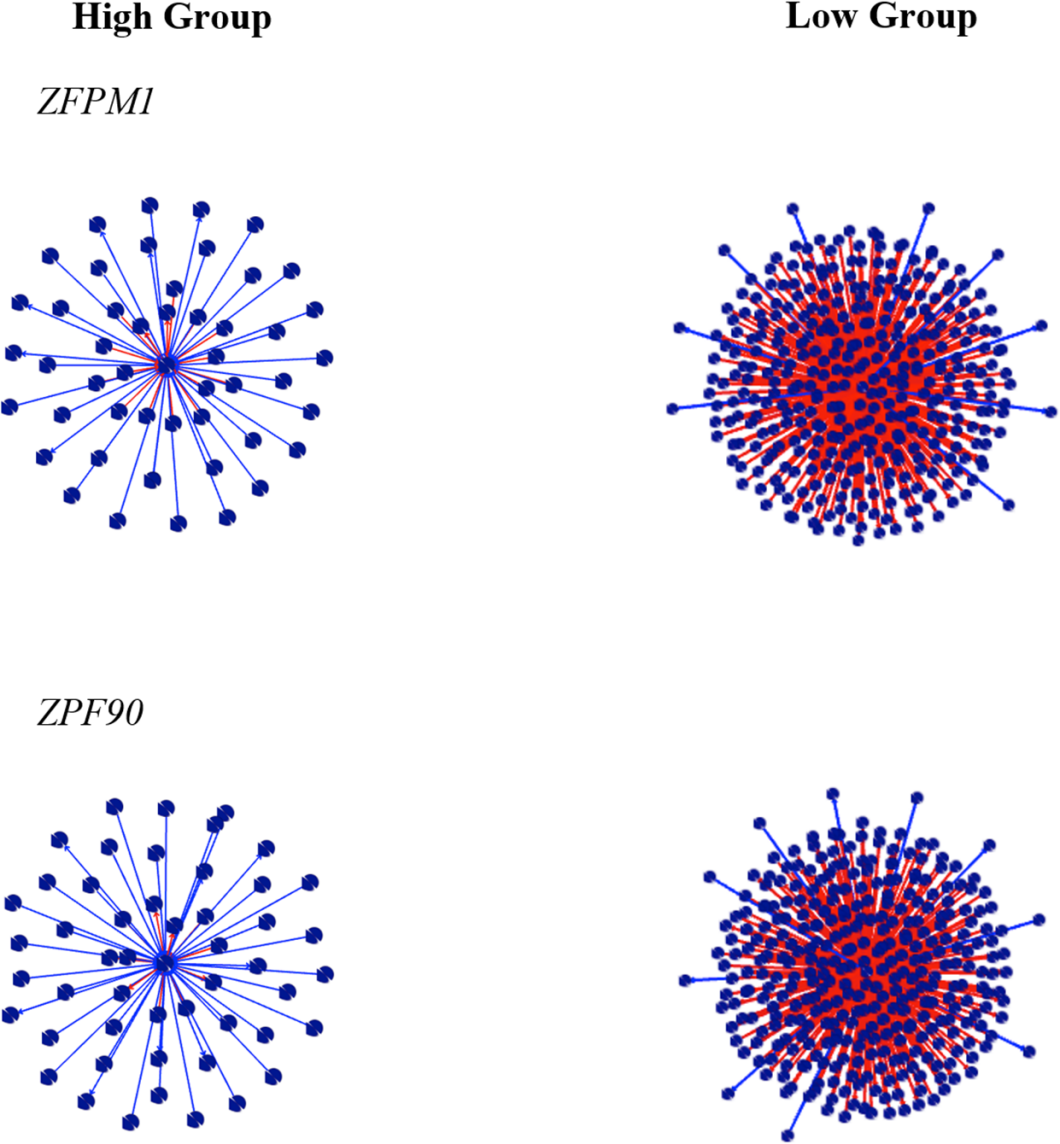
Negative differential hubbing (DH) between the high and low groups for IMF GEBV. The center spot represents the gene with high value of DH, the red edges represent the positive DH and blue edges represent the negative DH. Other spots represent the connections.

The top ten negative and positive DH values are shown in Tables 4 and 5, respectively. All DH results are presented in S4 Table. The modified version of the PCIT algorithm used in this study allowed all transcripts to be tested as putative regulators genes without prior knowledge of their function [10]. Among the top ten positive DH, three have been previously reported as associated with adipogenesis and adipose metabolism: *ANKRD26*, *HOXC5* and *PPAPDC2* (Fig 2). Among the top ten negative DH, two were identified by literature as good putative regulatory genes: Zinc finger protein, friend of GATA (FOG) family member 1 (*ZFPM1*) and zinc finger protein 90 (*ZFP90*) (Fig 3).

**Table 4:** Top 10 negative differentially hubbed (DH) genes comparing H and L groups of GEBV for IMF.

Ensembl Gene ID	Ensembl Transcript ID	Gene Symbol	DH	Transcript Biotype
ENSBTAG00000006639	ENSBTAT00000008722	*CSTF1*	-216	protein_coding
ENSBTAG00000022922	ENSBTAT00000055503	*ANKRD26*	-216	protein_coding
ENSBTAG00000018343	ENSBTAT00000024407	*NCR3*	-210	protein_coding
ENSBTAG00000006990	ENSBTAT00000009187	*MYRF*	-207	protein_coding
ENSBTAG00000025809	ENSBTAT00000036501	*ABHD8*	-204	protein_coding
ENSBTAG00000015208	ENSBTAT00000061091	*LRRC16B*	-204	protein_coding
ENSBTAG00000009769	ENSBTAT00000012885	Novel gene	-200	processed_pseudogene
ENSBTAG00000009634	ENSBTAT00000012696	*HOXC5*	-199	protein_coding
ENSBTAG00000011050	ENSBTAT00000014674	*PPAPDC2*	-198	protein_coding
ENSBTAG00000022922	ENSBTAT00000031151	*ANKRD26*	-198	protein_coding

**Table 5:** Top 10 positive differentially hubbed (DH) genes comparing H and L groups of GEBV for IMF.

Ensembl Gene ID	Ensembl Transcript ID	Gene Symbol	DH	Transcript Biotype
ENSBTAG00000007531	ENSBTAT00000009911	*NCF4*	302	protein_coding
ENSBTAG00000015766	ENSBTAT00000047968	*ZFPM1*	297	protein_coding
ENSBTAG00000006638	ENSBTAT00000008723	*BCL2L12*	296	protein_coding
ENSBTAG00000011590	ENSBTAT00000015396	*GLIPR1*	293	protein_coding
ENSBTAG00000025398	ENSBTAT00000035669	Novel gene	292	Novel protein coding
ENSBTAG00000007488	ENSBTAT00000032517	*ZPF90*	285	protein_coding
ENSBTAG00000004206	ENSBTAT00000005510	*LRRC55*	285	protein_coding
ENSBTAG00000014491	ENSBTAT00000019267	*ATP6V1G2*	282	protein_coding
UNANNOTATED	CUFF.25603.1	*-*	278	-
ENSBTAG00000002823	ENSBTAT00000003653	*MPZL1*	277	protein_coding

### Regulatory impact factor and phenotypic impact factor

The DH approach is not applicable to assess the importance of each DEG on differences in phenotype, so Hudson, Reverter and Dalrymple [11] proposed a new metric approach called “phenotypic impact factor” (PIF). PIF is based on DEG numerical properties, which “weigh” the contribution of the DEG based on the per unit differences in phenotype across groups of phenotypically extreme individuals. These authors also proposed the “regulatory impact factor” (RIF) to have a metric that accounts for changes in the molecular wiring of networks represented by changes in gene-gene correlation as well as changes in gene expression and PIF in response to changes in regulators expression level. Two types of RIF scores were developed. The RIF1 score prioritizes regulators that have a greater impact on the changes in wiring (i.e. correlation) in the network, whereas RIF2 score prioritizes regulators whose changes in expression mostly reflect the changes in expression of DEG. A complete list of all RIF1, RIF2 and PIF results are presented in S5, S6 e S7 Tables, respectively. A list of the top 10 RIF1 and RIF2 genes are presented in Tables 6 and 7, respectively.

**Table 6:** Top 10 genes identified by Regulatory Impact Factor 1 (RIF1) score by contrasting high minus low IMF groups.

Ensembl Gene ID	Ensembl Transcript ID	Gene Symbol	RIF1* ^,^^	Transcript Biotype
**Top Positive RIF1**				
ENSBTAG00000047048	ENSBTAT00000063303	Novel gene	9.27	protein coding
ENSBTAG00000010148	ENSBTAT00000013392	Novel gene	8.88	processed_pseudogene
ENSBTAG00000046926	ENSBTAT00000063044	GPX4	7.92	protein_coding
ENSBTAG00000047278	ENSBTAT00000063308	NDUFB11	7.71	protein_coding
ENSBTAG00000046850	ENSBTAT00000065286	Novel gene	7.64	pseudogene
**Top Negative RIF1**				
ENSBTAG00000011682	ENSBTAT00000015510	GLI2	-1.34	protein_coding
ENSBTAG00000001574	ENSBTAT00000002064	GPATCH2	-1.31	protein_coding
ENSBTAG00000019283	ENSBTAT00000025675	FAM186B	-1.31	protein_coding
ENSBTAG00000013066	ENSBTAT00000044139	IGF2	-1.31	protein_coding
ENSBTAG00000037440	ENSBTAT00000055187	Novel gene	-1.30	protein coding

*Note: RIF1 scores are presented as Z score normalized values.

**Table 7:** Top 10 genes identified by Regulatory Impact Factor 2 (RIF2) score by contrasting high minus low IMF groups.

Ensembl Gene ID	Ensembl Transcript ID	Gene Symbol	RIF2*	Transcript Biotype
		**Top Positive RIF1**		
ENSBTAG00000016997	ENSBTAT00000022603	LOC511180	7.60	protein coding
ENSBTAG00000015371	ENSBTAT00000020429	GABPB2	7.19	protein coding
ENSBTAG00000047712	ENSBTAT00000065359	Novel gene	6.75	processed pseudogene
ENSBTAG00000027879	ENSBTAT00000040201	MPC1	6.71	protein coding
ENSBTAG00000006398	ENSBTAT00000008388	TOMM7	6.70	protein coding
		**Top Negative RIF2**		
ENSBTAG00000030592	ENSBTAT00000031821	UBL5	-10.27	protein coding
ENSBTAG00000012803	ENSBTAT00000017007	SRP19	-10.19	protein coding
ENSBTAG00000007199	ENSBTAT00000009472	LOC529535	-8.34	pseudogene
ENSBTAG00000047194	ENSBTAT00000063248	Novel gene	-8.30	protein coding
ENSBTAG00000019949	ENSBTAT00000026574	DBR1	-8.13	protein coding

*Note: RIF2 scores are presented as Z score normalized values.

The RIF1 analysis identified many novel or pseudo genes as potential regulators that lead to differences between the low and high IMF groups. Two particularly interesting regulators included *GLI2* and *IGF2*. The RIF2 score identified additional candidate regulators, including two strong candidates: *MPC1* and *UBL5*. The PIF analysis identified many small RNAs as having a large impact on the IMF phenotype. In fact, 9 of the top 10 most highly ranked PIF genes are small RNAs.

To further investigate the functional associations of the 3,000 genes with higher scores of PIF, the functional term enrichment at DAVID database (http://www.david.abcc.ncifcrf.gov) was performed (S8 Table). Several significant clusters were enriched (BH-adj < 0.10), including GO terms of biological processes related to translation, generation of precursor metabolites and energy, ATP synthesis coupled proton transport, tricarboxylic acid cycle, acetyl-CoA catabolic process, acetyl-CoA metabolic process, posttranscriptional regulation of gene expression, protein catabolic process, mRNA metabolic process, RNA processing, RNA splicing, intracellular protein transport, intracellular transport and protein folding.

## Discussion

Intramuscular fat (IMF) quantity is an economically important trait, which influences the sensorial and nutritional value of meat. In addition, it is related to insulin resistance, which is an important predictive factor for disorders, such as cardiovascular disease, obesity and type 2 diabetes in human. In beef cattle, adipose tissue development is of significant interest because the deposition and composition of IMF are involved with organoleptic characteristics, consumer preference, public health, and producer profitability. Fat deposition is a consequence of the balance between energy intake and energy expenditure [26], which involves complex biological processes. Adipogenesis depends on a cascade of transcriptional factors activation [27] and events that are still unclear in species such as ruminants. As reviewed by Cristancho and Lazar [28] the adipogenesis process can be divided into three phases: commitment, transition and terminal differentiation. The commitment phase involves the conversion of mesenchymal stem cells (MSCs) to committed white preadipocytes, when occurs a dramatic alteration in cell shape mediated by extracellular matrix (ECM) factors. The committed white preadipocyte then goes through an epigenomic transition phase, when adipogenic stimuli (such as insulin and glucocorticoids) stimulates transcription factors that will induce peroxisome proliferator-activated receptor gamma (*PPARg*). In the terminal differentiation phase, committed white adipocyte becomes mature white adipocyte by induction of metabolic genes involved with triacylglycerol synthesis and degradation [28].

The present transcriptome study performed two strategies to identify differentially expressed genes (DEG), biological pathways and putative regulatory factors. The first strategy identified DEG between two groups (H and L) and biological pathways [18]. The second strategy identified putative regulatory factors and pathways [11]. In the first strategy, 77 significantly DEG between the groups were identified (S1 Table). It should be noted that in this study, the myofibers were not separated from intramuscular fat. Therefore, it is not possible to determine if the differential expression observed is the direct result of differences in the content of intramuscular fat or if there is an actual change in gene expression in either just the myofibers or intramuscular fat. Some of the genes and pathways herein identified agree with previous gene expression studies in preadipocyte differentiation process [29] and in IMF in cattle [30, 31].

The animals of low GEBV IMF group presented higher expression levels of genes associated with the first phase of adipocyte differentiation (MSC to committed white adipocytes). Some of these genes (*ROCK2*, *SPARC*, *ELN*, *RGS16*, *TGM2*, and *FLNA)* are involved in actinomyosin cytoskeleton remodeling, controlling the expression of adipogenic WNT genes [28]. Secreted protein acidic and rich in cysteine (*SPARC*) was the first matricellular protein to be linked to the accumulation of white adipose tissue [32]. *SPARC* inhibits differentiation of preadipocytes into adipocytes, by favoring osteoblastocyte differentiation. In this study the expression level of *SPARC* was higher in the lower GEBV IMF cattle, similar to that reported by Nie and Sage [33] in mice.

Animals with high GEBV IMF showed higher expression level of HIV-1 tat interactive protein 2 (*HTATIP2*) and signal transducer and activator of transcription 5A (*STAT5A*), which participate in molecular processes during the epigenomic transition phase, when insulin and glucocorticoids can lead to changes in the chromatin conformation, inducing the DNase I hypersensitivity “hotspots” [34]. In these “hotspots” Mandrup and collaborators [35] identified transcription factor motifs and binding of CCAAT-enhancer-binding proteins (*C/EBP*), glucocorticoid receptor (*GR*) and retinoid X receptor (*RXR*) and signal transducer and activator of transcription. *STAT5* is activated by kinases associated with transmembrane receptors and play role in cell growth and division, apoptosis and inflammation [36]. *STAT5* mediates energy homeostasis in response to endogenous cytokines and herein showed greater expression level in the group with high GEBV for IMF. *HTATIP2*, also more expressed in high GEBV for IMF, acts as a redox sensor, which is linked to regulation of nuclear import and is important in the transition phase [37].

Previously, Wang and collaborators [31] used spotted microarray to identify DEG due to different intramuscular fat content in Wagyu x Hereford and Piedmontese x Hereford crossbreds (*Bos taurus*). Similar to this study (Table 2), they identified DEG with the over-represented GO terms: muscle development and extracellular structure. Among the DEG identified by Wang and collaborators were *SPARC*, *RGS* and *STAT* family genes, which also were identified in this study. Lee and collaborators [30] evaluated gene expression due to differing IMF levels in *Bos taurus coreanae* animals and identified GO terms associated with biological adhesion, ECM, metabolic and development processes, which corroborate the findings of this study (Table 2). Lee and collaborators [30] identified some protein components of ECM (ITGA1, ITGB1, COL2A1, COL11A1, and COL11A2) significantly up-regulated in IMF, as identified herein.

In this study, *PPARg* and fatty acid synthase (*FAS*), major genes associated with terminal differentiation and fat deposition, presented low expression level in both H and L groups (S2 Table) and the difference of expression level was not significant between these groups. This could be explained by the fact that the animals used in the current study were in early stage of fat deposition (between 1.6 and 4.6% of IMF), while studies that identified terminal differentiation genes employed animal with 7.08% of IMF [38]. These findings corroborate with previous studies, which demonstrated that some *Bos taurus* breeds are genetically predisposed to deposit intramuscular fat earlier than *Bos indicus* breeds [32, 39, 40]. Lee and collaborators [31] evaluated differences in gene expression between three different adipose tissues (omental, subcutaneous and intramuscular). *FASN*, *FABP4*, *LPL*, *THRSP*, *DGAT1* and *PPARg*, all involved in adipogenesis and lipid metabolism, were significantly down-regulated in the intramuscular fat tissue as compared to other tissues. Based on these results, the authors suggested that those genes showed lower metabolic activities in intramuscular tissue of animals over 30 months old. Previous study [41] also reported that the *PPARg* expression level was different in the *Longissimus* muscle from Holstein and Charolais bulls slaughtered at 18 mo of age.

Similar to Huff and collaborators [42], De Jager and collaborators [6], presented the correlation between the *PPARg* gene set (*TF*, *ACSS2*, *ACLY*, *PPARg*, *CEBPA* and *CYB5A*) and IMF in Wagyu x Hereford and Piedmontese x Hereford (*Bos taurus* crosses) crosses, and Brahman (*Bos indicus*). These authors reported that the correlation between IMF percentage and *PPARg* gene set was weak in animal with lower IMF percentage (1.9% in average), i.e. in animals of Brahman breed.

Enrichment analyses detected 13 over-represented functional classes and pathways related to: 1) Small molecule signaling (L-cysteine, Zn^2+^, H_2_O_2_, Mg^2+^, ATP, retinoic acid, NO, IL-1ß, NF-kB and ROS); 2) Complex molecules (oxidized LDL); 3) Functional classes (inflammatory cytokines and protein tyrosin-kynase) as central regulators, which are involved in molecular mechanisms of adipogenesis (Table 3).

The L-cysteine pathway (Fig 1) regulates the expression of *ELN* and *MT3* that are involved in ECM process during the commitment phase of adipogenesis and have higher expression in animals with low GEBV for IMF. L-cysteine is involved in acylation, a covalent modification of intracellular polypeptides by the addition of C16 palmitic acid by a thioester linkage to cysteine residues of proteins [42, 43].

Elastin (*ELN*) protein is a component of the extracellular matrix (ECM) and with others EMC components (collagen and thrombospondin) are required for the expansion of fat mass process in obese animals [44]. Metallothionein-3 (*MT3*) is a zinc-binding protein and was observed that in null *MT3* male mice there is an increase in weight, resulting in obesity [45]. This obesity was caused by reduced energy expenditure and not from increased feed intake. This result suggests that *MT3* expression is negatively associated with fat variation, and agrees with our observation that animals in the low GEBV for IMF group show high expression level of *MT3*. On the other hand, phosphoglucomutase 1 (*PGM1*), which is involved with glycogen storage and is associated with the transition phase of adipogenesis, had higher expression in the animals with high GEBV for IMF. *PGM1* is associated with diseases such as hypertension, obesity and cardiovascular disease. It is overexpressed in skeletal muscle from insulin resistant humans [46]. The higher expression of *PGM1* in animals with in high IMF GEBV corroborates the findings reported by Nguyen and collaborators [46] in human skeletal muscle from obese individuals.

The retinoic acid and inflammatory cytokine pathways contain several genes that are in common, such as *STAT5A*, *ELN*, transglutaminase 2 (*TGM2*) and solute carrier family 2 (facilitated glucose transporter) member 4 (*SLC2A4*, also called *GLUT4*). These genes are involved in different phases of adipogenesis. Glucose transporter 4 (*GLUT4*) is a major regulator of glucose uptake in adipocytes (insulin regulation), which participates the adipogenic stimulation process. In mice with selective reduction of *GLUT4* there is a reduction in insulin-stimulated glucose uptake in adipocytes and consequently insulin resistance [47]. In this study, *GLUT4* had higher expression level in animal with low GEBV for IMF. The pathways identified in this study agree with previously published studies that showed the influence of retinoic acid and inflammatory cytokine systems on transcription process of peroxisome proliferator-activated receptor (PPAR) family, which regulate the PPARs expression level in human, mouse and ruminant [48].

The second strategy (PCIT, RIF1, RIF2 and PIF) was performed to better understand the correlations between expressed genes and to identify putative regulators of adipogenesis. These methods revealed regulators known to be involved in adipose development, obesity and retinoic acid signaling.

Differential hubbing (DH) or differential connectivity (DC) is the difference in the number of connections, measured as partial correlations a gene has in two different states. Previous studies discovered that a gene list of extreme values of DH showed higher percentage of transcriptional regulator factors than a list obtained by differential expression (DE) analysis [11]. In this study, the DH analysis using PCIT identified a host of interesting candidate regulators of IMF variation. Biologically interesting regulators include: ankyrin repeat domain 26 (*ANKRD26*) and phosphatidic acid phosphatase type 2 domain containing 2 (*PPAPDC2*), homeobox genes, such as *HOXC5*, zinc finger protein, friend of GATA (FOG) family member 1 (*ZFPM1*), and zinc finger protein 90 (*ZFP90)* as putative causal regulatory genes.

Ankyrin repeat domain 26 (*ANKRD26*) is expressed in the hypothalamus, brain, liver, adipose tissue and skeletal muscle and is located within the cell membrane. In humans and in mice, *ANKRD26* is responsible for white adipose tissue insulin response and appetite control [49]. Interestingly, one of the pathways enriched in this study, retinoic acid, has been demonstrated to be associated with adipose metabolism and Sahab and collaborators [50] showed that down-regulation of RAR could impact the expression of *ANKRD26*.

The homeobox family of genes is a conserved family of transcription factors, which play important roles in morphogenesis, metabolism [51] and differentiation of adipocytes [52].

Phosphatidic acid phosphatase type 2 domain containing 2 (*PPAPDC2*) is located within the endoplasmic reticulum and nuclear envelope in mammalian cells. Phosphatidic acid is a new class of lipid mediators, which are involved in in cell growth, proliferation and reproduction pathways such as a regulatory factor [53]. In *Saccharomyces cerevisiae*, phosphatidic acid was reported as an essential metabolic intermediate and a signaling lipid [54].

Zinc-finger protein, friend of *GATA* (FOG) family member 1 (*ZFPM1*) and zinc finger protein 90 (*ZFP90*) are important putative regulator genes in lipid metabolism. *GATA* family proteins are zinc-finger transcription factors, which recognize *GATA* motifs in DNA. *GATA* plays an important role in transcriptional regulation of many genes [55]. *ZFP90* is associated with RNA polymerase II core, which is involved in negative regulation of transcription (GO:0001078). Previous study also showed the zinc-finger protein as transcriptional regulators comparing the expression profiles between adipogenic and non-adipogenic fibroblasts [28].

The RIF1 analysis identified as putative regulators *IGF2* and *GLI2*, and RIF2 identified *MPC1* and UBL5. The identification of *IGF2* as a potential regulator of the degree of IMF has been suggested previously in pigs [56] and in mice [57], as it is involved in adipocyte proliferation [58]. The *GLI2* gene, although not documented to play a role in adipose development, is a regulator of cellular proliferation and is known to be regulated by retinoic acid signaling [59], which is consistent with the signaling mechanisms identified by Pathway Studio. The *MPC1* gene is a part of a mitochondrial pyruvate carrier complex and has been implicated in the mitochondrial response to insulin and *PPARg* signaling [60]. The *UBL5* gene has been suggested to impact adipose deposition in both the pig and human. The *UBL5* is a candidate gene for meat quality and IMF in the pig [61] and also associated with body fat and fat accumulation related to metabolic dysfunction and diabetes in humans [62, 63]. Previous study also reported that PPARg proteasomal degradation is ubiquitin-dependent and the stable overexpression of ubiquitin gene reduces *PPARg* protein levels and suppress adipocyte differentiation in human cell [64].

One of the top PIF genes was microRNA-1281, previously identified as an adipose expressed miRNA that may regulate the lipid metabolism gene *EP300* [65]. The functional enrichment by DAVID database showed that the PIF approach was well suited to identify putative regulators involved with the phenotype studied (intramuscular fat variation).

Associations between GEBVs and RNA abundance are based on the assumption that changes in RNA levels are related to genetic differences in IMF potential in animals with extreme GEBVs for IMF since environmental variation known to impact IMF is account for within the genetic prediction model. However, it is possible that environmental factors could still impact which RNAs are identified as differentially expressed.

In this study, changes in gene expression in this study may indicate changes in IMF, muscle or other cell types within muscle that impact the content of IMF. A portion of the DE genes identified in this study may be related to differences in the lipid content within intramuscular adipocytes or extracellular matrix (ECM) deposition. Changes in genes related to ECM proteins deposition in this study are consistent with previous studies that indicate that increased ECM proteins deposition is associated with increased lipid content within intramuscular adipocytes [66, 67]. This may provide an explanation for DE genes related to ECM growth.

The genes identified by association and network analysis in this study could be related to any of the physiological processes involved in creating variation in IMF. It is difficult to determine if IMF deposition was altered versus other processes like lipid filling in intramuscular adipocytes or other changes in muscle physiology since IMF deposition rates were not measured over time in this study. The lack of FA and TAG synthesis pathway genes may indicate that the DE genes in this study may be related to the lipid content or filling of intramuscular adipocytes which results in the observed variation in IMF levels. Since this study provides novel information about transcriptional differences in the Nellore *Bos indicus* breed, it is possible that novel genes may be detected that impact the variation in IMF levels. This may represent genetic or physiological differences in Nellore compared to other breeds. Further studies are needed to discern the difference between these two possible explanations.

## Conclusions

The present study showed the complexity of *Longissimus dorsi* muscle transcriptome and the molecular mechanisms involved in lipid metabolism related to differences in IMF in extreme IMF GEBV Nellore cattle. Differential gene expression and pathway enrichment analysis identified a number of genes and pathways related to adipogenesis and lipid metabolism. These changes in gene expression and associated pathways indicate that animals in the high GEBV for IMF mature earlier in respect to IMF content. At the same age, animals with low GEBV for IMF had a higher expression of genes related to the commitment phase of adipogenesis, whereas animal with high GEBV for IMF had higher expression of genes related to the transition phase. Furthermore, there appears to be differences between *Bos indicus* and *Bos taurus* in regards of IMF deposition. This study indicated that retinoic acid signaling, *IGF2* and *ANKRD26* are important regulators of molecular mechanisms related to IMF content and adipogenesis process. These findings contribute to a better understanding of the molecular mechanisms underlying fat deposition and energy balance in muscle of ruminant, and may provide important information to other species, such as human and mouse.

## Supporting Information

S1 Fig Scatter plot between intramuscular fat percentage and genomic estimated breeding value (GEBV) from *Longissimus dorsi* muscle of Nellore steers. Y-axis represents the genomic estimated breeding value (GEBV) and X-axis represents intramuscular fat percentage.
(DOCX)

S2 Fig Empirical and fitted dispersion values plotted against the mean of the normalized counts from RNA-Seq data of *Longissimus dorsi* muscle of Nellore steers. The black dots represent the empirical dispersion values and the red line represents fitted dispersion values (log_2_). Y-axis represents the dispersion of expression level and X-axis represents the mean of normalized counts.
(DOCX)

S3 Fig Plot of normalized mean versus log2 fold change to contrast Low and High groups based on genomic estimated breeding values (GEBV) for intramuscular fat (IMF) percentage. Red points represent significant (10% FDR) genes.
(DOCX)

S4 Fig Histogram of p-values from RNA-Seq data of *Longissimus dorsi* muscle of Nellore steers by DESeq program. Y-axis represents the frequency of p-values and X-axis representes the residual of p-values.
(DOCX)

S5 Fig Retinoic acid pathway genes identified as differentially expressed between the high and low groups for IMF GEBV are shown here (FDR ≤ 0.10, adjusted for multiple testing using Benjamini-Hochberg method). The genes shown in red had higher expression in the low IMF group and those in blue had higher expression in animals from the High IMF group.
(DOCX)

S6 Fig Inflammatory cytokine pathway genes identified as differentially expressed between the high and low groups for IMF GEBV are shown here (FDR ≤ 0.10, adjusted for multiple testing using Benjamini-Hochberg method). The genes shown in red had higher expression in the low IMF group and those in blue had higher expression in animals from the High IMF group.
(DOCX)

S1 Table Backfat thickness (mm), ribeye area (cm2) and genomic estimated breeding values (GEBV). (XLSX)

S2 Table The differentially expressed genes (DGE) obtained between High and Low groups based on genomic estimated breeding values (GEBV) for intramuscular fat (IMF) percentage in Nellore steers with FDR < 10%. (DOCX)

S3 Table Expression level of all genes identified in the transcriptome analysis from Longissimus dorsi muscle of Nellore steers. (XLSX)

S4 Table Differentially hubbing genes comparing High and Low groups based on genomic estimated breeding values (GEBV) for intramuscular fat (IMF) percentage. (XLSX)

S5 Table Regulatory impact factor 1 (RIF) scores between the high and low IMF groups in Nellore steers. (XLSX)

S6 Table Regulatory impact factor 2 (RIF) scores between the high and low IMF groups in Nellore steers. (XLSX)

S7 Table Phenotypic impact factor (PIF) scores between high and low IMF groups in Nellore steers. (XLSX)

S8 Table Functional term enrichment (FDR < 0.01) of 3,000 genes with higher phenotypic impact factor (PIF) scores comparing high and low IMF GEBV in Nellore steers. (DOCX)
