# Supplementary material for: Putative Regulatory Factors Associated with Intramuscular Fat Content
Source: PLoS One. 2015 Jun 4;10(6):e0128350. doi: 10.1371/journal.pone.0128350 (PMC4456163; doi:10.1371/journal.pone.0128350)
Supplement: S1 Fig — Y-axis represents the genomic estimated breeding value (GEBV) and X-axis represents intramuscular fat percentage. (DOCX) [file pone.0128350.s001.docx]

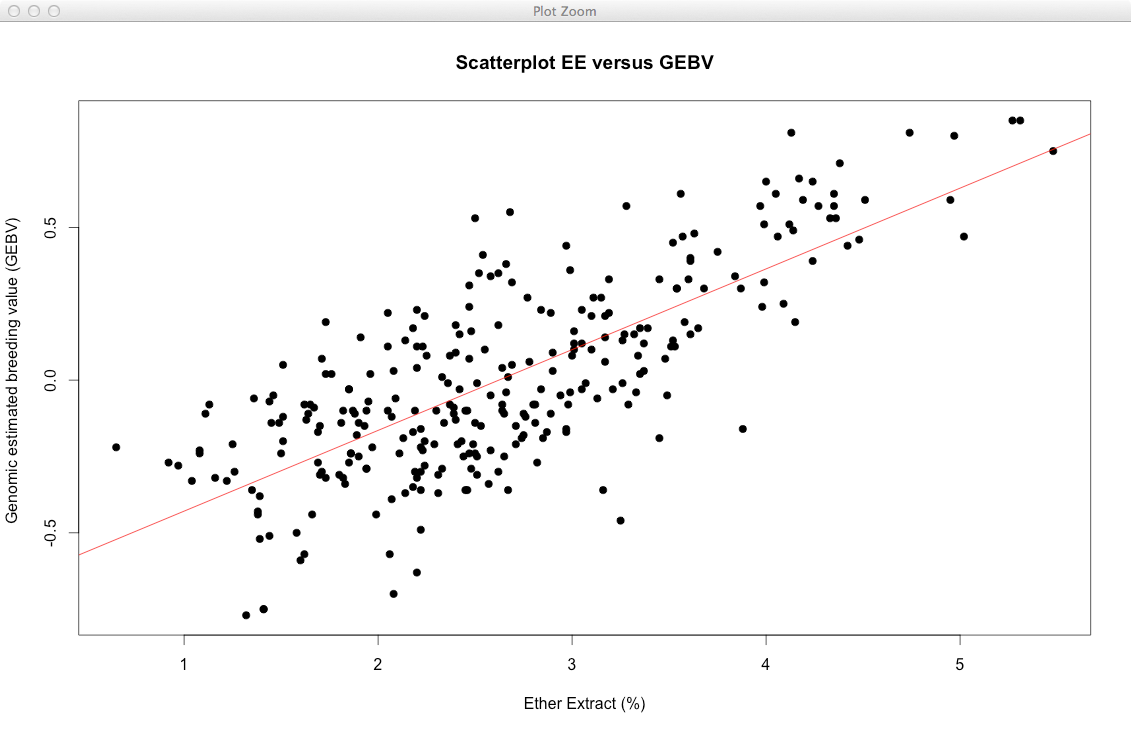


Intramuscular fat percentage

Genomic estimated breeding value (GEBV)

Figure S1. Scatter plot between intramuscular fat percentage and genomic estimated breeding value (GEBV) from *Longissimus dorsi* muscle of Nellore steers. Y-axis represents the genomic estimated breeding value (GEBV) and X-axis represents intramuscular fat percentage.
