# Supplementary material for: Putative Regulatory Factors Associated with Intramuscular Fat Content
Source: PLoS One. 2015 Jun 4;10(6):e0128350. doi: 10.1371/journal.pone.0128350 (PMC4456163; doi:10.1371/journal.pone.0128350)
Supplement: S2 Fig — The black dots represent the empirical dispersion values and the red line represents fitted dispersion values (log2). Y-axis represents the dispersion of expression level and X-axis represents the mean of normalized counts. (DOCX) [file pone.0128350.s002.docx]

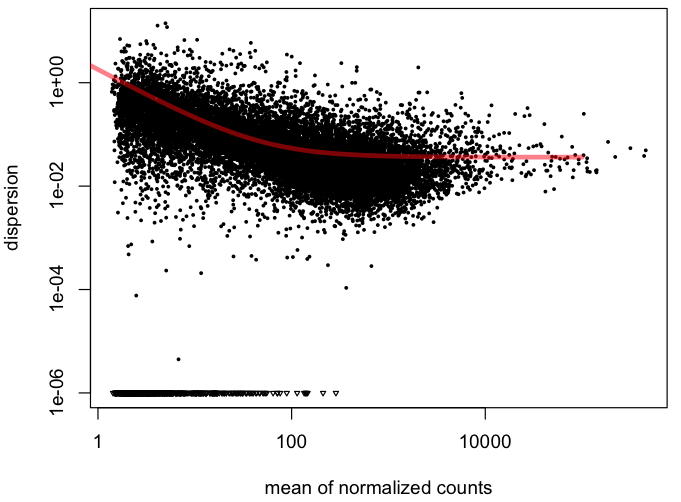


Figure S2: Empirical (black dots) and fitted (red lines) dispersion values (log_2_) plotted against the mean of the normalized counts from RNA-Seq data of *Longissimus dorsi* muscle of Nellore steers. The black dots represent the empirical dispersion values and the red line represents fitted dispersion values (log_2_). Y-axis represents the dispersion of expression level and X-axis represents the mean of normalized counts.
