# Supplementary material for: Putative Regulatory Factors Associated with Intramuscular Fat Content
Source: PLoS One. 2015 Jun 4;10(6):e0128350. doi: 10.1371/journal.pone.0128350 (PMC4456163; doi:10.1371/journal.pone.0128350)
Supplement: S3 Fig — Red points represent significant (10% FDR) genes. (DOCX) [file pone.0128350.s003.docx]

Figure S3. Plot of normalized mean versus log2 fold change to contrast Low and High groups based on genomic estimated breeding values (GEBV) for intramuscular fat (IMF) percentage. Red points represent significant (10% FDR) genes.
