# Supplementary material for: Putative Regulatory Factors Associated with Intramuscular Fat Content
Source: PLoS One. 2015 Jun 4;10(6):e0128350. doi: 10.1371/journal.pone.0128350 (PMC4456163; doi:10.1371/journal.pone.0128350)
Supplement: S4 Fig — Y-axis represents the frequency of p-values and X-axis representes the residual of p-values. (DOCX) [file pone.0128350.s004.docx]

Figure S4. Histogram of p -values from RNA-Seq data of *Longissimus dorsi* muscle of Nellore steers by DESeq program. Y-axis represents the frequency of p-values and X-axis representes the residual of p-values.
