# Supplementary material for: Putative Regulatory Factors Associated with Intramuscular Fat Content
Source: PLoS One. 2015 Jun 4;10(6):e0128350. doi: 10.1371/journal.pone.0128350 (PMC4456163; doi:10.1371/journal.pone.0128350)
Supplement: S6 Fig — The genes shown in red had higher expression in the low IMF group and those in blue had higher expression in animals from the High IMF group. (DOCX) [file pone.0128350.s006.docx]

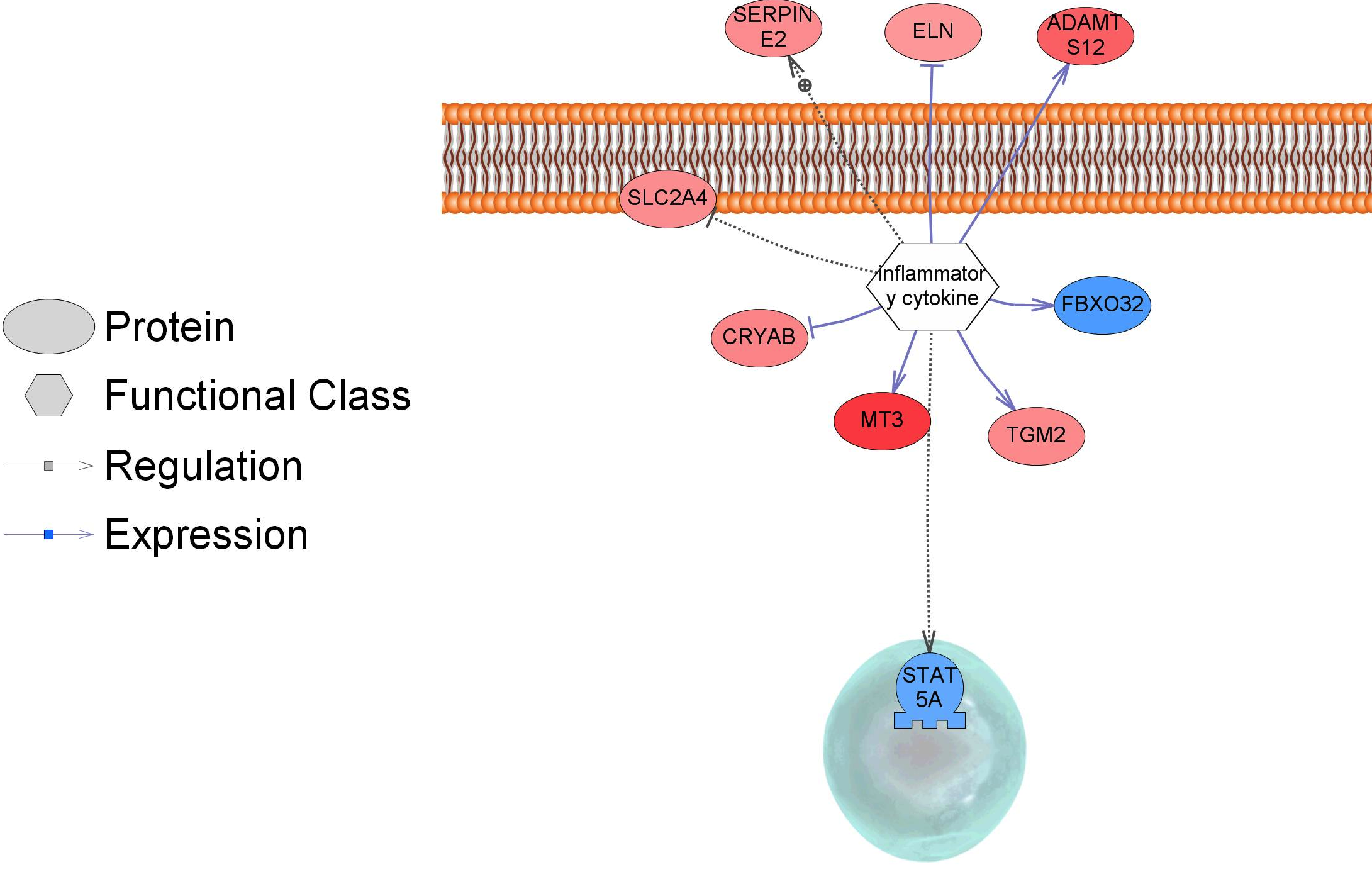


Figure S6. Inflammatory cytokine pathway genes identified as differentially expressed between the high and low groups for IMF GEBV are shown here (FDR ≤ 0.10, adjusted for multiple testing using Benjamini-Hochberg method). The genes shown in red had higher expression in the low IMF group and those in blue had higher expression in animals from the High IMF group.
