# Supplementary material for: Putative Regulatory Factors Associated with Intramuscular Fat Content
Source: PLoS One. 2015 Jun 4;10(6):e0128350. doi: 10.1371/journal.pone.0128350 (PMC4456163; doi:10.1371/journal.pone.0128350)
Supplement: S2 Table — (DOCX) [file pone.0128350.s008.docx]

Table S2. The differentially expressed genes (DGE) obtained between High and Low groups based on genomic estimated breeding values (GEBV) for intramuscular fat (IMF) percentage in Nellore steers with FDR < 10%.

| **Ensembl Gene ID** | **Gene Symbol** | **baseMean^1^** | **baseMeanA^2^** | **baseMeanB^3^** | **foldChange^4^** | **pval** | **padj** |
| --- | --- | --- | --- | --- | --- | --- | --- |
| ENSBTAG00000021508 | *LMOD3* | 7,928.17 | 10,274.52 | 5,581.83 | 0.54 | 5.46x10^-11^ | 8.78x10^-7^ |
| ENSBTAG00000025210 | *COL4A2* | 9,959.52 | 7,174.80 | 12,744.25 | 1.78 | 7.36x10^-10^ | 5.92x10^-6^ |
| ENSBTAG00000012849 | *COL4A1* | 18,562.12 | 13,722.93 | 23,401.32 | 1.71 | 7.64x10^-9^ | 4.10x10^-5^ |
| ENSBTAG00000011465 | *MYBPH* | 98.28 | 44.10 | 152.46 | 3.46 | 5.97x10^-8^ | 2.40x10^-4^ |
| ENSBTAG00000005102 | *PHTF2* | 1,177.63 | 1,464.31 | 890.95 | 0.61 | 1.10x10^-7^ | 3.53x10^-4^ |
| ENSBTAG00000015303 | *MPP6* | 339.00 | 426.21 | 251.79 | 0.59 | 5.18x10^-7^ | 1.39x10^-3^ |
| ENSBTAG00000010389 | *STBD1* | 1,501.74 | 1,940.19 | 1,063.28 | 0.55 | 9.24x10^-7^ | 2.12x10^-3^ |
| ENSBTAG00000014835 | *SPARC* | 20,870.41 | 16,334.19 | 25,406.64 | 1.56 | 1.32x10^-6^ | 2.66x10^-3^ |
| ENSBTAG00000014885 | *MYOM3* | 9,806.49 | 7,226.13 | 12,386.86 | 1.71 | 3.02x10^-6^ | 5.40x10^-3^ |
| ENSBTAG00000001183 | *KLHL33* | 2,297.48 | 2,775.51 | 1,819.45 | 0.66 | 3.77x10^-6^ | 6.01x10^-3^ |
| ENSBTAG00000009145 | *SLC7A4* | 110.63 | 152.73 | 68.52 | 0.45 | 4.11x10^-6^ | 6.01x10^-3^ |
| ENSBTAG00000024555 | *EFCAB2* | 112.00 | 144.62 | 79.37 | 0.55 | 5.19x10^-6^ | 6.97x10^-3^ |
| ENSBTAG00000018382 | *F1ME02* | 564.14 | 449.52 | 678.75 | 1.51 | 1.74x10^-5^ | 2.15x10^-2^ |
| ENSBTAG00000000828 | *CAPN6* | 893.42 | 723.26 | 1,063.58 | 1.47 | 2.47x10^-5^ | 2.72x10^-2^ |
| ENSBTAG00000013419 | *HTATIP2* | 282.52 | 353.88 | 211.17 | 0.60 | 2.81x10^-5^ | 2.72x10^-2^ |
| ENSBTAG00000016709 | *NT5C3* | 407.17 | 491.36 | 322.98 | 0.66 | 2.67x10^-5^ | 2.72x10^-2^ |
| ENSBTAG00000004347 | *GPR116* | 3,229.34 | 2,623.20 | 3,835.49 | 1.46 | 2.87x10^-5^ | 2.72x10^-2^ |
| ENSBTAG00000004036 | *GJC1* | 106.13 | 70.93 | 141.32 | 1.99 | 3.46x10^-5^ | 2.87x10^-2^ |
| ENSBTAG00000004126 | *MLF1* | 2,367.33 | 2,924.83 | 1,809.84 | 0.62 | 3.87x10^-5^ | 2.87x10^-2^ |
| ENSBTAG00000022989 | *FAM174B* | 75.66 | 98.74 | 52.58 | 0.53 | 4.07x10^-5^ | 2.87x10^-2^ |
| ENSBTAG00000006819 | *CTAGE5* | 1,023.75 | 1,223.21 | 824.29 | 0.67 | 4.10x10^-5^ | 2.87x10^-2^ |
| ENSBTAG00000018775 | *TPX2* | 440.69 | 547.59 | 333.78 | 0.61 | 3.29x10^-5^ | 2.87x10^-2^ |
| ENSBTAG00000038141 | *F1MKM4* | 216.29 | 161.28 | 271.29 | 1.68 | 3.59x10^-5^ | 2.87x10^-2^ |
| ENSBTAG00000019658 | *ASB16* | 2,684.07 | 3,179.60 | 2,188.53 | 0.69 | 4.57x10^-5^ | 2.97x10^-2^ |
| ENSBTAG00000001514 | *ASB11* | 946.09 | 1,127.32 | 764.86 | 0.68 | 4.95x10^-5^ | 2.97x10^-2^ |
| ENSBTAG00000013750 | *B3GALNT2* | 528.29 | 426.39 | 630.19 | 1.48 | 4.98x10^-5^ | 2.97x10^-2^ |
| ENSBTAG00000035844 | *HRASLS* | 1,279.90 | 1,578.56 | 981.24 | 0.62 | 4.77x10^-5^ | 2.97x10-^2^ |
| ENSBTAG00000014863 | *GYPC* | 2,021.53 | 2,405.81 | 1,637.26 | 0.68 | 5.28x10^-5^ | 3.30x10^-2^ |
| ENSBTAG00000007211 | *ASB12* | 3,772.06 | 4,750.26 | 2,793.86 | 0.59 | 5.72x10^-5^ | 3.18x10^-2^ |
| ENSBTAG00000016194 | *FBXO32* | 3,310.54 | 4,176.72 | 2,444.36 | 0.59 | 6.10x10^-5^ | 3.27x10^-2^ |
| ENSBTAG00000011190 | *FLNA* | 6,136.61 | 4,791.10 | 7,482.11 | 1.56 | 7.46x10^-5^ | 3.87x10^-2^ |
| ENSBTAG00000019052 | *ANK3* | 473.36 | 561.03 | 385.70 | 0.69 | 7.84x10^-5^ | 3.94x10^-2^ |
| ENSBTAG00000002391 | *TGFB1I1* | 326.41 | 254.27 | 398.56 | 1.57 | 1.00x10^-4^ | 4.25x10^-2^ |
| ENSBTAG00000002914 | *GALNTL4* | 132.92 | 97.19 | 168.66 | 1.74 | 9.83x10^-5^ | 4.25x10^-2^ |
| ENSBTAG00000011869 | *CSRP3* | 29,763.36 | 18,942.70 | 40,584.02 | 2.14 | 9.95x10^-5^ | 4.25x10^-2^ |
| ENSBTAG00000009496 | *STAT5A* | 393.40 | 469.54 | 317.25 | 0.68 | 9.73x10^-5^ | 4.25x10^-2^ |
| ENSBTAG00000019517 | *ELN* | 2,071.46 | 1,645.83 | 2,497.09 | 1.52 | 9.48x10^-5^ | 4.25x10^-2^ |
| ENSBTAG00000012505 | *ARHGEF17* | 420.66 | 333.77 | 507.55 | 1.52 | 9.02x10^-5^ | 4.25x10^-2^ |
| ENSBTAG00000016525 | *F1MMN6* | 767.64 | 615.74 | 919.54 | 1.49 | 1.12x10^-4^ | 4.64x10^-2^ |
| ENSBTAG00000002126 | *PFKFB2* | 388.79 | 455.85 | 321.73 | 0.71 | 1.21x10^-4^ | 4.89x10^-2^ |
| ENSBTAG00000018747 | *PRKAA2* | 711.69 | 839.81 | 583.57 | 0.69 | 1.29x10^-4^ | 4.98x10^-2^ |
| ENSBTAG00000020764 | *CNN2* | 156.65 | 118.37 | 194.92 | 1.65 | 1.30x10^-4^ | 4.98x10^-2^ |
| ENSBTAG00000012558 | *ADAMTS12* | 173.39 | 111.41 | 235.37 | 2.11 | 1.55x10^-4^ | 5.57x10^-2^ |
| ENSBTAG00000013632 | *GRM4* | 36.44 | 50.18 | 22.71 | 0.45 | 1.55x10^-4^ | 5.57x10^-2^ |
| ENSBTAG00000031134 | [*F1MPT3*](http://www.uniprot.org/uniprot/F1MPT3) | 950.01 | 771.75 | 1,128.27 | 1.46 | 1.56x10^-4^ | 5.57x10^-2^ |
| ENSBTAG00000004305 | *RGS16* | 108.28 | 76.15 | 140.40 | 1.84 | 1.77x10^-4^ | 6.21x10^-2^ |
| ENSBTAG00000011913 | *CKAP4* | 178.87 | 140.93 | 216.80 | 1.54 | 1.88x10^-4^ | 6.40x10^-2^ |
| ENSBTAG00000007632 | *FAM151B* | 168.02 | 205.93 | 130.11 | 0.63 | 1.91x10^-4^ | 6.40x10^-2^ |
| ENSBTAG00000014930 | *MYLK2* | 18,138.96 | 22,394.85 | 13,883.08 | 0.62 | 2.16x10^-4^ | 7.09x10^-2^ |
| ENSBTAG00000003836 | *ADAM19* | 1,291.98 | 1,086.20 | 1,497.77 | 1.38 | 2.35x10^-4^ | 7.11x10^-2^ |
| ENSBTAG00000004570 | *PRRX1* | 272.28 | 201.58 | 342.98 | 1.70 | 2.22x10^-4^ | 7.11x10^-2^ |
| ENSBTAG00000017233 | *RNF213* | 125.78 | 79.82 | 171.75 | 2.15 | 2.32x10^-4^ | 7.11x10^-2^ |
| ENSBTAG00000016918 | *MYOF* | 595.62 | 485.32 | 705.92 | 1.45 | 2.38x10^-4^ | 7.11x10^-2^ |
| ENSBTAG00000005932 | *FAM184B* | 31.60 | 42.34 | 20.86 | 0.49 | 2.34x10^-4^ | 7.11x10^-2^ |
| ENSBTAG00000012450 | *RAPGEF2* | 955.88 | 784.17 | 1,127.59 | 1.44 | 2.45x10^-4^ | 7.18x10^-2^ |
| ENSBTAG00000016013 | *ASB14* | 858.94 | 1,017.39 | 700.48 | 0.69 | 2.57x10^-4^ | 7.38x10^-2^ |
| ENSBTAG00000021768 | *E1BIN6* | 336.38 | 420.58 | 252.19 | 0.60 | 2.64x10^-4^ | 7.45x10^-2^ |
| ENSBTAG00000004259 | *HPCAL1* | 256.20 | 202.46 | 309.94 | 1.53 | 2.70x10^-4^ | 7.49x10^-2^ |
| ENSBTAG00000006253 | *FLNC* | 113,919.49 | 78,869.35 | 148,969.62 | 1.89 | 2.88x10^-4^ | 7.66x10^-2^ |
| ENSBTAG00000016208 | *TGM2* | 1,430.90 | 1,083.10 | 1,778.69 | 1.64 | 2.90x10^-4^ | 7.66x10^-2^ |
| ENSBTAG00000046162 | *ZNF385C* | 58.42 | 36.78 | 80.06 | 2.18 | 2.90x10^-4^ | 7.66x10^-2^ |
| ENSBTAG00000025099 | *RNF145* | 470.56 | 388.72 | 552.40 | 1.42 | 3.04x10^-4^ | 7.90x10^-2^ |
| ENSBTAG00000010940 | *HSPB7* | 6,297.66 | 4,989.43 | 7,605.90 | 1.52 | 3.18x10^-4^ | 8.13x10^-2^ |
| ENSBTAG00000011010 | *PRND* | 38.92 | 15.31 | 62.54 | 4.08 | 3.32x10^-4^ | 8.21x10^-2^ |
| ENSBTAG00000012720 | *ANKRD2* | 4,559.69 | 2,834.86 | 6,284.53 | 2.22 | 3.31x10^-4^ | 8.21x10^-2^ |
| ENSBTAG00000005729 | *FBXL4* | 721.12 | 869.82 | 572.42 | 0.66 | 3.61x10^-4^ | 8.40x10^-2^ |
| ENSBTAG00000010179 | *COL5A3* | 2,843.18 | 2,391.27 | 3,295.09 | 1.38 | 3.60x10^-4^ | 8.40x10^-2^ |
| ENSBTAG00000011324 | *EMILIN1* | 736.34 | 606.71 | 865.98 | 1.43 | 3.55x10^-4^ | 8.40x10^-2^ |
| ENSBTAG00000019090 | *PLEKHH3* | 584.98 | 680.38 | 489.57 | 0.72 | 3.66x10^-4^ | 8.40x10^-2^ |
| ENSBTAG00000027051 | *PTAFR* | 56.15 | 39.30 | 73.01 | 1.86 | 3.69x10^-4^ | 8.40x10^-2^ |
| ENSBTAG00000048210 | *G5E6M6* | 485.58 | 605.60 | 365.57 | 0.60 | 3.70x10^-4^ | 8.40x10^-2^ |
| ENSBTAG00000006679 | *MITF* | 632.94 | 750.45 | 515.42 | 0.69 | 3.98x10^-4^ | 8.79x10^-2^ |
| ENSBTAG00000013922 | *MOSPD1* | 332.11 | 451.56 | 212.66 | 0.47 | 3.96x10^-4^ | 8.79x10^-2^ |
| ENSBTAG00000013131 | *FAM110D* | 59.37 | 40.81 | 77.93 | 1.91 | 4.10x10^-4^ | 8.92x10^-2^ |
| ENSBTAG00000019011 | *PGM1* | 587.89 | 781.26 | 394.53 | 0.50 | 4.19x10^-4^ | 9.00x10^-2^ |
| ENSBTAG00000020097 | *PERP* | 15.84 | 7.90 | 23.78 | 3.01 | 4.40x10^-4^ | 9.33x10^-2^ |
| ENSBTAG00000022920 | *RNF128* | 725.41 | 852.37 | 598.45 | 0.70 | 4.47x10-4 | 9.35x10^-2^ |

^1^ baseMean - mean normalised counts, averaged over all samples from both groups (H and L).

^2^ baseMeanA mean normalised counts from high group.

^3^ baseMeanB - mean normalised counts from low group.

^4^ foldChange - fold change from low to high group.

^5^ pval - p value for the statistical significance of this change.

^6^ padj - p value adjusted for multiple testing with the Benjamini-Hochberg procedure, which controls false discovery rate (FDR).
