# Supplementary material for: Putative Regulatory Factors Associated with Intramuscular Fat Content
Source: PLoS One. 2015 Jun 4;10(6):e0128350. doi: 10.1371/journal.pone.0128350 (PMC4456163; doi:10.1371/journal.pone.0128350)
Supplement: S8 Table — (DOCX) [file pone.0128350.s014.docx]

Table S8. Functional term enrichment (FDR < 0.01) of 3,000 genes with higher phenotypic impact factor (PIF) scores comparing high and low IMF GEBV in Nellore steers.

| **Category** | **Term** | **Count^1^** | **%^2^** | **P-Value** | **Benjamini** |
| --- | --- | --- | --- | --- | --- |
| SP_PIR_KEYWORDS | [acetylation](http://www.uniprot.org/keywords/?query=acetylation) | 587 | 21.2 | 7.3E-118 | 3.4E-115 |
| GOTERM_CC_FAT | [ribonucleoprotein complex](http://www.ebi.ac.uk/QuickGO/GTerm?id=GO:0030529) | 194 | 7.0 | 3.2E-60 | 1.7E-57 |
| GOTERM_BP_FAT | [translation](http://www.ebi.ac.uk/QuickGO/GTerm?id=GO:0006412) | 164 | 5.9 | 1.3E-59 | 3.5E-56 |
| SP_PIR_KEYWORDS | [ribonucleoprotein](http://www.uniprot.org/keywords/?query=ribonucleoprotein) | 144 | 5.2 | 3.8E-53 | 8.8E-51 |
| GOTERM_CC_FAT | [mitochondrion](http://www.ebi.ac.uk/QuickGO/GTerm?id=GO:0005739) | 289 | 10.4 | 2.5E-49 | 6.6E-47 |
| KEGG_PATHWAY | [Ribosome](http://david.abcc.ncifcrf.gov/kegg.jsp?path=bta03010$Ribosome&termId=470015024&source=kegg) | 77 | 2.8 | 1.3E-48 | 2.1E-46 |
| SP_PIR_KEYWORDS | [ribosomal protein](http://www.uniprot.org/keywords/?query=ribosomal%20protein) | 118 | 4.3 | 4.1E-46 | 6.3E-44 |
| GOTERM_CC_FAT | [ribosome](http://www.ebi.ac.uk/QuickGO/GTerm?id=GO:0005840) | 125 | 4.5 | 7.1E-45 | 1.3E-42 |
| GOTERM_MF_FAT | [structural constituent of ribosome](http://www.ebi.ac.uk/QuickGO/GTerm?id=GO:0003735) | 105 | 3.8 | 4.7E-42 | 4.4E-39 |
| SP_PIR_KEYWORDS | [phosphoprotein](http://www.uniprot.org/keywords/?query=phosphoprotein) | 651 | 23.5 | 8.8E-40 | 1.0E-37 |
| GOTERM_CC_FAT | [mitochondrial part](http://www.ebi.ac.uk/QuickGO/GTerm?id=GO:0044429) | 176 | 6.3 | 2.9E-39 | 3.8E-37 |
| SP_PIR_KEYWORDS | [cytoplasm](http://www.uniprot.org/keywords/?query=cytoplasm) | 409 | 14.7 | 3.2E-35 | 3.0E-33 |
| SP_PIR_KEYWORDS | [mitochondrion](http://www.uniprot.org/keywords/?query=mitochondrion) | 226 | 8.1 | 1.2E-33 | 9.2E-32 |
| GOTERM_BP_FAT | [generation of precursor metabolites and energy](http://www.ebi.ac.uk/QuickGO/GTerm?id=GO:0006091) | 108 | 3.9 | 2.0E-32 | 2.7E-29 |
| GOTERM_CC_FAT | [organelle envelope](http://www.ebi.ac.uk/QuickGO/GTerm?id=GO:0031967) | 160 | 5.8 | 2.0E-30 | 2.2E-28 |
| GOTERM_CC_FAT | [envelope](http://www.ebi.ac.uk/QuickGO/GTerm?id=GO:0031975) | 160 | 5.8 | 2.9E-30 | 2.6E-28 |
| GOTERM_CC_FAT | [mitochondrial membrane](http://www.ebi.ac.uk/QuickGO/GTerm?id=GO:0031966) | 130 | 4.7 | 5.9E-30 | 4.5E-28 |
| GOTERM_CC_FAT | [mitochondrial envelope](http://www.ebi.ac.uk/QuickGO/GTerm?id=GO:0005740) | 132 | 4.8 | 9.1E-29 | 5.3E-27 |
| GOTERM_CC_FAT | [mitochondrial inner membrane](http://www.ebi.ac.uk/QuickGO/GTerm?id=GO:0005743) | 116 | 4.2 | 8.9E-29 | 5.9E-27 |
| GOTERM_CC_FAT | [organelle inner membrane](http://www.ebi.ac.uk/QuickGO/GTerm?id=GO:0019866) | 117 | 4.2 | 2.1E-28 | 1.1E-26 |
| GOTERM_CC_FAT | [organelle membrane](http://www.ebi.ac.uk/QuickGO/GTerm?id=GO:0031090) | 199 | 7.2 | 6.5E-28 | 3.1E-26 |
| GOTERM_CC_FAT | [intracellular non-membrane-bounded organelle](http://www.ebi.ac.uk/QuickGO/GTerm?id=GO:0043232) | 335 | 12.1 | 6.5E-26 | 2.6E-24 |
| GOTERM_CC_FAT | [non-membrane-bounded organelle](http://www.ebi.ac.uk/QuickGO/GTerm?id=GO:0043228) | 335 | 12.1 | 6.5E-26 | 2.6E-24 |
| GOTERM_CC_FAT | [intracellular organelle lumen](http://www.ebi.ac.uk/QuickGO/GTerm?id=GO:0070013) | 204 | 7.4 | 6.1E-26 | 2.7E-24 |
| GOTERM_CC_FAT | [membrane-enclosed lumen](http://www.ebi.ac.uk/QuickGO/GTerm?id=GO:0031974) | 210 | 7.6 | 7.1E-26 | 2.7E-24 |
| GOTERM_CC_FAT | [organelle lumen](http://www.ebi.ac.uk/QuickGO/GTerm?id=GO:0043233) | 204 | 7.4 | 7.8E-26 | 2.8E-24 |
| GOTERM_BP_FAT | [protein catabolic process](http://www.ebi.ac.uk/QuickGO/GTerm?id=GO:0030163) | 114 | 4.1 | 2.7E-25 | 2.4E-22 |
| SP_PIR_KEYWORDS | [transit peptide](http://www.uniprot.org/keywords/?query=transit%20peptide) | 143 | 5.2 | 5.7E-24 | 3.8E-22 |
| GOTERM_BP_FAT | [proteolysis involved in cellular protein catabolic process](http://www.ebi.ac.uk/QuickGO/GTerm?id=GO:0051603) | 106 | 3.8 | 6.5E-24 | 4.4E-21 |
| GOTERM_BP_FAT | [cellular macromolecule catabolic process](http://www.ebi.ac.uk/QuickGO/GTerm?id=GO:0044265) | 117 | 4.2 | 1.3E-23 | 5.1E-21 |
| GOTERM_BP_FAT | [cellular protein catabolic process](http://www.ebi.ac.uk/QuickGO/GTerm?id=GO:0044257) | 106 | 3.8 | 9.6E-24 | 5.2E-21 |
| GOTERM_BP_FAT | [protein localization](http://www.ebi.ac.uk/QuickGO/GTerm?id=GO:0008104) | 156 | 5.6 | 1.2E-23 | 5.3E-21 |
| GOTERM_BP_FAT | [macromolecule catabolic process](http://www.ebi.ac.uk/QuickGO/GTerm?id=GO:0009057) | 126 | 4.5 | 2.7E-23 | 9.1E-21 |
| KEGG_PATHWAY | [Oxidative phosphorylation](http://david.abcc.ncifcrf.gov/kegg.jsp?path=bta00190$Oxidative%20phosphorylation&termId=470014953&source=kegg) | 73 | 2.6 | 1.7E-22 | 1.4E-20 |
| GOTERM_BP_FAT | [protein transport](http://www.ebi.ac.uk/QuickGO/GTerm?id=GO:0015031) | 144 | 5.2 | 7.4E-23 | 2.2E-20 |
| GOTERM_BP_FAT | [establishment of protein localization](http://www.ebi.ac.uk/QuickGO/GTerm?id=GO:0045184) | 144 | 5.2 | 9.7E-23 | 2.6E-20 |
| GOTERM_MF_FAT | [structural molecule activity](http://www.ebi.ac.uk/QuickGO/GTerm?id=GO:0005198) | 137 | 4.9 | 6.3E-23 | 2.9E-20 |
| GOTERM_MF_FAT | [RNA binding](http://www.ebi.ac.uk/QuickGO/GTerm?id=GO:0003723) | 127 | 4.6 | 1.6E-22 | 4.8E-20 |
| KEGG_PATHWAY | [Huntington's disease](http://david.abcc.ncifcrf.gov/kegg.jsp?path=bta05016$Huntington's%20disease&termId=470015105&source=kegg) | 85 | 3.1 | 1.0E-21 | 5.7E-20 |
| GOTERM_BP_FAT | [modification-dependent macromolecule catabolic process](http://www.ebi.ac.uk/QuickGO/GTerm?id=GO:0043632) | 96 | 3.5 | 2.6E-21 | 6.4E-19 |
| GOTERM_BP_FAT | [modification-dependent protein catabolic process](http://www.ebi.ac.uk/QuickGO/GTerm?id=GO:0019941) | 96 | 3.5 | 2.6E-21 | 6.4E-19 |
| KEGG_PATHWAY | [Parkinson's disease](http://david.abcc.ncifcrf.gov/kegg.jsp?path=bta05012$Parkinson's%20disease&termId=470015103&source=kegg) | 69 | 2.5 | 3.2E-20 | 1.4E-18 |
| KEGG_PATHWAY | [Spliceosome](http://david.abcc.ncifcrf.gov/kegg.jsp?path=bta03040$Spliceosome&termId=470015029&source=kegg) | 67 | 2.4 | 5.7E-20 | 1.9E-18 |
| KEGG_PATHWAY | [Alzheimer's disease](http://david.abcc.ncifcrf.gov/kegg.jsp?path=bta05010$Alzheimer's%20disease&termId=470015102&source=kegg) | 78 | 2.8 | 9.8E-20 | 2.8E-18 |
| SP_PIR_KEYWORDS | [mitochondrion inner membrane](http://www.uniprot.org/keywords/?query=mitochondrion%20inner%20membrane) | 80 | 2.9 | 9.3E-20 | 5.4E-18 |
| GOTERM_BP_FAT | [intracellular transport](http://www.ebi.ac.uk/QuickGO/GTerm?id=GO:0046907) | 104 | 3.7 | 7.4E-18 | 1.7E-15 |
| UP_SEQ_FEATURE | transit peptide:Mitochondrion | 140 | 5.0 | 6.9E-19 | 1.8E-15 |
| SP_PIR_KEYWORDS | [protein biosynthesis](http://www.uniprot.org/keywords/?query=protein%20biosynthesis) | 51 | 1.8 | 3.7E-17 | 1.9E-15 |
| KEGG_PATHWAY | [Proteasome](http://david.abcc.ncifcrf.gov/kegg.jsp?path=bta03050$Proteasome&termId=470015030&source=kegg) | 34 | 1.2 | 1.3E-16 | 2.7E-15 |
| GOTERM_CC_FAT | [respiratory chain](http://www.ebi.ac.uk/QuickGO/GTerm?id=GO:0070469) | 43 | 1.6 | 1.1E-16 | 3.4E-15 |
| GOTERM_CC_FAT | [proteasome complex](http://www.ebi.ac.uk/QuickGO/GTerm?id=GO:0000502) | 36 | 1.3 | 5.8E-17 | 3.7E-15 |
| SP_PIR_KEYWORDS | [isopeptide bond](http://www.uniprot.org/keywords/?query=isopeptide%20bond) | 62 | 2.2 | 1.2E-16 | 5.1E-15 |
| SP_PIR_KEYWORDS | [rna-binding](http://www.uniprot.org/keywords/?query=rna-binding) | 87 | 3.1 | 7.4E-16 | 3.3E-14 |
| GOTERM_BP_FAT | [electron transport chain](http://www.ebi.ac.uk/QuickGO/GTerm?id=GO:0022900) | 48 | 1.7 | 2.6E-16 | 4.6E-14 |
| SP_PIR_KEYWORDS | [respiratory chain](http://www.uniprot.org/keywords/?query=respiratory%20chain) | 47 | 1.7 | 1.8E-15 | 6.8E-14 |
| GOTERM_CC_FAT | [mitochondrial membrane part](http://www.ebi.ac.uk/QuickGO/GTerm?id=GO:0044455) | 34 | 1.2 | 2.1E-14 | 6.1E-13 |
| GOTERM_MF_FAT | [translation factor activity, nucleic acid binding](http://www.ebi.ac.uk/QuickGO/GTerm?id=GO:0008135) | 40 | 1.4 | 6.0E-15 | 1.4E-12 |
| SP_PIR_KEYWORDS | [proteasome](http://www.uniprot.org/keywords/?query=proteasome) | 35 | 1.3 | 4.0E-14 | 1.4E-12 |
| UP_SEQ_FEATURE | cross-link:Glycyl lysine isopeptide (Lys-Gly) (interchain with G-Cter in ubiquitin) | 47 | 1.7 | 1.1E-15 | 1.4E-12 |
| GOTERM_CC_FAT | [nuclear lumen](http://www.ebi.ac.uk/QuickGO/GTerm?id=GO:0031981) | 138 | 5.0 | 6.8E-14 | 1.9E-12 |
| GOTERM_CC_FAT | [ribosomal subunit](http://www.ebi.ac.uk/QuickGO/GTerm?id=GO:0033279) | 34 | 1.2 | 1.2E-13 | 3.2E-12 |
| GOTERM_CC_FAT | [mitochondrial matrix](http://www.ebi.ac.uk/QuickGO/GTerm?id=GO:0005759) | 55 | 2.0 | 1.5E-13 | 3.7E-12 |
| GOTERM_CC_FAT | [mitochondrial lumen](http://www.ebi.ac.uk/QuickGO/GTerm?id=GO:0031980) | 55 | 2.0 | 1.5E-13 | 3.7E-12 |
| GOTERM_BP_FAT | [mRNA metabolic process](http://www.ebi.ac.uk/QuickGO/GTerm?id=GO:0016071) | 65 | 2.3 | 4.7E-14 | 9.1E-12 |
| GOTERM_BP_FAT | [cellular macromolecule localization](http://www.ebi.ac.uk/QuickGO/GTerm?id=GO:0070727) | 80 | 2.9 | 6.8E-14 | 1.2E-11 |
| GOTERM_BP_FAT | [cellular protein localization](http://www.ebi.ac.uk/QuickGO/GTerm?id=GO:0034613) | 79 | 2.8 | 1.6E-13 | 2.7E-11 |
| GOTERM_BP_FAT | [intracellular protein transport](http://www.ebi.ac.uk/QuickGO/GTerm?id=GO:0006886) | 75 | 2.7 | 2.9E-13 | 4.5E-11 |
| GOTERM_CC_FAT | [cytosol](http://www.ebi.ac.uk/QuickGO/GTerm?id=GO:0005829) | 98 | 3.5 | 2.7E-12 | 6.4E-11 |
| SP_PIR_KEYWORDS | [protein transport](http://www.uniprot.org/keywords/?query=protein%20transport) | 89 | 3.2 | 2.7E-12 | 8.8E-11 |
| GOTERM_BP_FAT | [RNA splicing](http://www.ebi.ac.uk/QuickGO/GTerm?id=GO:0008380) | 48 | 1.7 | 7.4E-13 | 1.1E-10 |
| GOTERM_BP_FAT | [mRNA processing](http://www.ebi.ac.uk/QuickGO/GTerm?id=GO:0006397) | 58 | 2.1 | 8.4E-13 | 1.2E-10 |
| GOTERM_BP_FAT | [ubiquitin-dependent protein catabolic process](http://www.ebi.ac.uk/QuickGO/GTerm?id=GO:0006511) | 46 | 1.7 | 1.1E-12 | 1.4E-10 |
| SP_PIR_KEYWORDS | [ubl conjugation](http://www.uniprot.org/keywords/?query=ubl%20conjugation) | 79 | 2.8 | 1.4E-11 | 4.0E-10 |
| SP_PIR_KEYWORDS | [electron transport](http://www.uniprot.org/keywords/?query=electron%20transport) | 47 | 1.7 | 1.3E-11 | 4.1E-10 |
| GOTERM_BP_FAT | [energy derivation by oxidation of organic compounds](http://www.ebi.ac.uk/QuickGO/GTerm?id=GO:0015980) | 37 | 1.3 | 3.7E-12 | 4.8E-10 |
| GOTERM_BP_FAT | [RNA processing](http://www.ebi.ac.uk/QuickGO/GTerm?id=GO:0006396) | 88 | 3.2 | 6.8E-12 | 8.4E-10 |
| GOTERM_MF_FAT | [nucleotide binding](http://www.ebi.ac.uk/QuickGO/GTerm?id=GO:0000166) | 347 | 12.5 | 5.8E-12 | 1.1E-9 |
| GOTERM_CC_FAT | [spliceosome](http://www.ebi.ac.uk/QuickGO/GTerm?id=GO:0005681) | 38 | 1.4 | 1.2E-10 | 2.8E-9 |
| GOTERM_BP_FAT | [oxidative phosphorylation](http://www.ebi.ac.uk/QuickGO/GTerm?id=GO:0006119) | 33 | 1.2 | 2.8E-11 | 3.2E-9 |
| GOTERM_MF_FAT | [translation initiation factor activity](http://www.ebi.ac.uk/QuickGO/GTerm?id=GO:0003743) | 28 | 1.0 | 2.8E-11 | 4.3E-9 |
| GOTERM_CC_FAT | [cytosolic part](http://www.ebi.ac.uk/QuickGO/GTerm?id=GO:0044445) | 34 | 1.2 | 2.3E-10 | 5.1E-9 |
| GOTERM_MF_FAT | [acid-amino acid ligase activity](http://www.ebi.ac.uk/QuickGO/GTerm?id=GO:0016881) | 49 | 1.8 | 6.0E-11 | 7.0E-9 |
| GOTERM_MF_FAT | [hydrogen ion transmembrane transporter activity](http://www.ebi.ac.uk/QuickGO/GTerm?id=GO:0015078) | 38 | 1.4 | 5.4E-11 | 7.3E-9 |
| GOTERM_CC_FAT | [nucleoplasm](http://www.ebi.ac.uk/QuickGO/GTerm?id=GO:0005654) | 90 | 3.2 | 3.6E-10 | 7.5E-9 |
| GOTERM_MF_FAT | [small conjugating protein ligase activity](http://www.ebi.ac.uk/QuickGO/GTerm?id=GO:0019787) | 38 | 1.4 | 2.1E-10 | 2.2E-8 |
| GOTERM_BP_FAT | [cellular respiration](http://www.ebi.ac.uk/QuickGO/GTerm?id=GO:0045333) | 28 | 1.0 | 2.0E-10 | 2.2E-8 |
| GOTERM_MF_FAT | [monovalent inorganic cation transmembrane transporter activity](http://www.ebi.ac.uk/QuickGO/GTerm?id=GO:0015077) | 38 | 1.4 | 3.2E-10 | 3.0E-8 |
| GOTERM_CC_FAT | [small ribosomal subunit](http://www.ebi.ac.uk/QuickGO/GTerm?id=GO:0015935) | 20 | 0.7 | 2.5E-9 | 5.1E-8 |
| SP_PIR_KEYWORDS | [ubl conjugation pathway](http://www.uniprot.org/keywords/?query=ubl%20conjugation%20pathway) | 73 | 2.6 | 2.0E-9 | 5.3E-8 |
| GOTERM_MF_FAT | [cytoskeletal protein binding](http://www.ebi.ac.uk/QuickGO/GTerm?id=GO:0008092) | 71 | 2.6 | 8.3E-10 | 7.0E-8 |
| SP_PIR_KEYWORDS | [transport](http://www.uniprot.org/keywords/?query=transport) | 225 | 8.1 | 2.9E-9 | 7.5E-8 |
| GOTERM_MF_FAT | [inorganic cation transmembrane transporter activity](http://www.ebi.ac.uk/QuickGO/GTerm?id=GO:0022890) | 43 | 1.6 | 1.8E-9 | 1.4E-7 |
| GOTERM_BP_FAT | [translational initiation](http://www.ebi.ac.uk/QuickGO/GTerm?id=GO:0006413) | 21 | 0.8 | 3.6E-9 | 3.9E-7 |
| GOTERM_MF_FAT | [ligase activity, forming carbon-nitrogen bonds](http://www.ebi.ac.uk/QuickGO/GTerm?id=GO:0016879) | 52 | 1.9 | 6.1E-9 | 4.4E-7 |
| SP_PIR_KEYWORDS | [oxidative phosphorylation](http://www.uniprot.org/keywords/?query=oxidative%20phosphorylation) | 26 | 0.9 | 2.5E-8 | 5.9E-7 |
| SP_PIR_KEYWORDS | [mrna splicing](http://www.uniprot.org/keywords/?query=mrna%20splicing) | 43 | 1.6 | 2.6E-8 | 5.9E-7 |
| GOTERM_BP_FAT | [protein modification by small protein conjugation or removal](http://www.ebi.ac.uk/QuickGO/GTerm?id=GO:0070647) | 31 | 1.1 | 6.5E-9 | 6.8E-7 |
| GOTERM_BP_FAT | [protein modification by small protein conjugation](http://www.ebi.ac.uk/QuickGO/GTerm?id=GO:0032446) | 28 | 1.0 | 1.1E-8 | 1.1E-6 |
| SP_PIR_KEYWORDS | [Initiation factor](http://www.uniprot.org/keywords/?query=Initiation%20factor) | 24 | 0.9 | 4.9E-8 | 1.1E-6 |
| KEGG_PATHWAY | [Ubiquitin mediated proteolysis](http://david.abcc.ncifcrf.gov/kegg.jsp?path=bta04120$Ubiquitin%20mediated%20proteolysis&termId=470015048&source=kegg) | 51 | 1.8 | 5.7E-8 | 1.2E-6 |
| KEGG_PATHWAY | [Citrate cycle (TCA cycle)](http://david.abcc.ncifcrf.gov/kegg.jsp?path=bta00020$Citrate%20cycle%20(TCA%20cycle)&termId=470014938&source=kegg) | 20 | 0.7 | 8.9E-8 | 1.7E-6 |
| GOTERM_BP_FAT | [protein folding](http://www.ebi.ac.uk/QuickGO/GTerm?id=GO:0006457) | 45 | 1.6 | 2.0E-8 | 2.0E-6 |
| SP_PIR_KEYWORDS | [methylation](http://www.uniprot.org/keywords/?query=methylation) | 50 | 1.8 | 9.7E-8 | 2.0E-6 |
| SP_PIR_KEYWORDS | [Hydrogen ion transport](http://www.uniprot.org/keywords/?query=Hydrogen%20ion%20transport) | 24 | 0.9 | 1.0E-7 | 2.1E-6 |
| SP_PIR_KEYWORDS | [mrna processing](http://www.uniprot.org/keywords/?query=mrna%20processing) | 47 | 1.7 | 1.1E-7 | 2.2E-6 |
| SP_PIR_KEYWORDS | [nucleotide-binding](http://www.uniprot.org/keywords/?query=nucleotide-binding) | 221 | 8.0 | 1.2E-7 | 2.2E-6 |
| SP_PIR_KEYWORDS | [membrane-associated complex](http://www.uniprot.org/keywords/?query=membrane-associated%20complex) | 23 | 0.8 | 1.5E-7 | 2.6E-6 |
| GOTERM_CC_FAT | [mitochondrial respiratory chain](http://www.ebi.ac.uk/QuickGO/GTerm?id=GO:0005746) | 15 | 0.5 | 1.4E-7 | 2.7E-6 |
| GOTERM_CC_FAT | [melanosome](http://www.ebi.ac.uk/QuickGO/GTerm?id=GO:0042470) | 28 | 1.0 | 1.5E-7 | 2.9E-6 |
| GOTERM_CC_FAT | [pigment granule](http://www.ebi.ac.uk/QuickGO/GTerm?id=GO:0048770) | 28 | 1.0 | 1.5E-7 | 2.9E-6 |
| GOTERM_MF_FAT | [rRNA binding](http://www.ebi.ac.uk/QuickGO/GTerm?id=GO:0019843) | 16 | 0.6 | 4.4E-8 | 2.9E-6 |
| GOTERM_MF_FAT | [unfolded protein binding](http://www.ebi.ac.uk/QuickGO/GTerm?id=GO:0051082) | 29 | 1.0 | 5.0E-8 | 3.1E-6 |
| GOTERM_MF_FAT | [purine ribonucleotide binding](http://www.ebi.ac.uk/QuickGO/GTerm?id=GO:0032555) | 272 | 9.8 | 5.4E-8 | 3.2E-6 |
| GOTERM_MF_FAT | [ribonucleotide binding](http://www.ebi.ac.uk/QuickGO/GTerm?id=GO:0032553) | 272 | 9.8 | 5.4E-8 | 3.2E-6 |
| GOTERM_CC_FAT | [proton-transporting two-sector ATPase complex](http://www.ebi.ac.uk/QuickGO/GTerm?id=GO:0016469) | 25 | 0.9 | 1.9E-7 | 3.5E-6 |
| SP_PIR_KEYWORDS | [Chaperone](http://www.uniprot.org/keywords/?query=Chaperone) | 46 | 1.7 | 2.4E-7 | 4.1E-6 |
| GOTERM_BP_FAT | [proton transport](http://www.ebi.ac.uk/QuickGO/GTerm?id=GO:0015992) | 25 | 0.9 | 4.5E-8 | 4.2E-6 |
| GOTERM_CC_FAT | [cytosolic ribosome](http://www.ebi.ac.uk/QuickGO/GTerm?id=GO:0022626) | 13 | 0.5 | 2.5E-7 | 4.4E-6 |
| GOTERM_CC_FAT | [nucleoplasm part](http://www.ebi.ac.uk/QuickGO/GTerm?id=GO:0044451) | 72 | 2.6 | 3.4E-7 | 5.7E-6 |
| GOTERM_CC_FAT | [internal side of plasma membrane](http://www.ebi.ac.uk/QuickGO/GTerm?id=GO:0009898) | 44 | 1.6 | 3.8E-7 | 6.3E-6 |
| GOTERM_BP_FAT | [hydrogen transport](http://www.ebi.ac.uk/QuickGO/GTerm?id=GO:0006818) | 25 | 0.9 | 7.4E-8 | 6.7E-6 |
| GOTERM_MF_FAT | [threonine-type peptidase activity](http://www.ebi.ac.uk/QuickGO/GTerm?id=GO:0070003) | 14 | 0.5 | 1.3E-7 | 6.9E-6 |
| GOTERM_MF_FAT | [threonine-type endopeptidase activity](http://www.ebi.ac.uk/QuickGO/GTerm?id=GO:0004298) | 14 | 0.5 | 1.3E-7 | 6.9E-6 |
| GOTERM_BP_FAT | [aerobic respiration](http://www.ebi.ac.uk/QuickGO/GTerm?id=GO:0009060) | 17 | 0.6 | 8.5E-8 | 7.4E-6 |
| GOTERM_MF_FAT | [purine nucleotide binding](http://www.ebi.ac.uk/QuickGO/GTerm?id=GO:0017076) | 280 | 10.1 | 1.4E-7 | 7.5E-6 |
| GOTERM_BP_FAT | [cell redox homeostasis](http://www.ebi.ac.uk/QuickGO/GTerm?id=GO:0045454) | 26 | 0.9 | 1.0E-7 | 8.6E-6 |
| GOTERM_CC_FAT | [proteasome core complex](http://www.ebi.ac.uk/QuickGO/GTerm?id=GO:0005839) | 14 | 0.5 | 5.8E-7 | 9.3E-6 |
| SP_PIR_KEYWORDS | [er-golgi transport](http://www.uniprot.org/keywords/?query=er-golgi%20transport) | 26 | 0.9 | 1.1E-6 | 1.8E-5 |
| GOTERM_CC_FAT | [cytosolic small ribosomal subunit](http://www.ebi.ac.uk/QuickGO/GTerm?id=GO:0022627) | 12 | 0.4 | 1.1E-6 | 1.8E-5 |
| GOTERM_CC_FAT | [proton-transporting ATP synthase complex](http://www.ebi.ac.uk/QuickGO/GTerm?id=GO:0045259) | 16 | 0.6 | 1.3E-6 | 2.0E-5 |
| GOTERM_MF_FAT | [oxidoreductase activity, acting on NADH or NADPH](http://www.ebi.ac.uk/QuickGO/GTerm?id=GO:0016651) | 23 | 0.8 | 4.5E-7 | 2.2E-5 |
| GOTERM_BP_FAT | [energy coupled proton transport, down electrochemical gradient](http://www.ebi.ac.uk/QuickGO/GTerm?id=GO:0015985) | 22 | 0.8 | 3.1E-7 | 2.6E-5 |
| GOTERM_BP_FAT | [ATP synthesis coupled proton transport](http://www.ebi.ac.uk/QuickGO/GTerm?id=GO:0015986) | 22 | 0.8 | 3.1E-7 | 2.6E-5 |
| GOTERM_MF_FAT | [transcription activator activity](http://www.ebi.ac.uk/QuickGO/GTerm?id=GO:0016563) | 39 | 1.4 | 5.5E-7 | 2.6E-5 |
| SP_PIR_KEYWORDS | [nad](http://www.uniprot.org/keywords/?query=nad) | 52 | 1.9 | 1.8E-6 | 2.8E-5 |
| SP_PIR_KEYWORDS | [cytoskeleton](http://www.uniprot.org/keywords/?query=cytoskeleton) | 72 | 2.6 | 2.5E-6 | 3.9E-5 |
| GOTERM_MF_FAT | [protein transporter activity](http://www.ebi.ac.uk/QuickGO/GTerm?id=GO:0008565) | 25 | 0.9 | 1.0E-6 | 4.3E-5 |
| GOTERM_MF_FAT | [actin binding](http://www.ebi.ac.uk/QuickGO/GTerm?id=GO:0003779) | 45 | 1.6 | 1.0E-6 | 4.4E-5 |
| SP_PIR_KEYWORDS | [nucleus](http://www.uniprot.org/keywords/?query=nucleus) | 335 | 12.1 | 3.6E-6 | 5.4E-5 |
| GOTERM_CC_FAT | [myofibril](http://www.ebi.ac.uk/QuickGO/GTerm?id=GO:0030016) | 23 | 0.8 | 4.1E-6 | 6.0E-5 |
| COG_ONTOLOGY | [Posttranslational modification, protein turnover, chaperones](http://www.ncbi.nlm.nih.gov/COG/new/) | 36 | 1.3 | 2.0E-6 | 7.1E-5 |
| SMART | [RRM](http://smart.embl.de/smart/do_annotation.pl?DOMAIN=SM00360) | 49 | 1.8 | 2.5E-7 | 7.3E-5 |
| GOTERM_CC_FAT | [contractile fiber](http://www.ebi.ac.uk/QuickGO/GTerm?id=GO:0043292) | 24 | 0.9 | 5.4E-6 | 7.8E-5 |
| GOTERM_MF_FAT | [GTP binding](http://www.ebi.ac.uk/QuickGO/GTerm?id=GO:0005525) | 76 | 2.7 | 2.0E-6 | 7.9E-5 |
| GOTERM_MF_FAT | [transcription factor binding](http://www.ebi.ac.uk/QuickGO/GTerm?id=GO:0008134) | 42 | 1.5 | 2.0E-6 | 8.1E-5 |
| SP_PIR_KEYWORDS | [threonine protease](http://www.uniprot.org/keywords/?query=threonine%20protease) | 14 | 0.5 | 5.9E-6 | 8.2E-5 |
| SP_PIR_KEYWORDS | [actin-binding](http://www.uniprot.org/keywords/?query=actin-binding) | 38 | 1.4 | 5.8E-6 | 8.3E-5 |
| GOTERM_BP_FAT | [protein targeting](http://www.ebi.ac.uk/QuickGO/GTerm?id=GO:0006605) | 33 | 1.2 | 1.1E-6 | 8.6E-5 |
| GOTERM_BP_FAT | [tricarboxylic acid cycle](http://www.ebi.ac.uk/QuickGO/GTerm?id=GO:0006099) | 14 | 0.5 | 1.1E-6 | 8.7E-5 |
| GOTERM_BP_FAT | [acetyl-CoA catabolic process](http://www.ebi.ac.uk/QuickGO/GTerm?id=GO:0046356) | 14 | 0.5 | 1.1E-6 | 8.7E-5 |
| GOTERM_CC_FAT | [eukaryotic translation initiation factor 3 complex](http://www.ebi.ac.uk/QuickGO/GTerm?id=GO:0005852) | 10 | 0.4 | 6.7E-6 | 9.3E-5 |
| GOTERM_BP_FAT | [ion transmembrane transport](http://www.ebi.ac.uk/QuickGO/GTerm?id=GO:0034220) | 22 | 0.8 | 1.3E-6 | 1.0E-4 |
| GOTERM_CC_FAT | [endomembrane system](http://www.ebi.ac.uk/QuickGO/GTerm?id=GO:0012505) | 87 | 3.1 | 7.5E-6 | 1.0E-4 |
| SP_PIR_KEYWORDS | [Spliceosome](http://www.uniprot.org/keywords/?query=Spliceosome) | 29 | 1.0 | 8.3E-6 | 1.1E-4 |
| GOTERM_BP_FAT | [vesicle-mediated transport](http://www.ebi.ac.uk/QuickGO/GTerm?id=GO:0016192) | 71 | 2.6 | 1.6E-6 | 1.2E-4 |
| SP_PIR_KEYWORDS | [rrna-binding](http://www.uniprot.org/keywords/?query=rrna-binding) | 11 | 0.4 | 9.1E-6 | 1.2E-4 |
| GOTERM_BP_FAT | [regulation of cellular component biogenesis](http://www.ebi.ac.uk/QuickGO/GTerm?id=GO:0044087) | 23 | 0.8 | 1.7E-6 | 1.2E-4 |
| GOTERM_BP_FAT | [acetyl-CoA metabolic process](http://www.ebi.ac.uk/QuickGO/GTerm?id=GO:0006084) | 14 | 0.5 | 2.5E-6 | 1.7E-4 |
| GOTERM_BP_FAT | [posttranscriptional regulation of gene expression](http://www.ebi.ac.uk/QuickGO/GTerm?id=GO:0010608) | 32 | 1.2 | 2.6E-6 | 1.8E-4 |
| GOTERM_MF_FAT | [guanyl ribonucleotide binding](http://www.ebi.ac.uk/QuickGO/GTerm?id=GO:0032561) | 76 | 2.7 | 5.3E-6 | 2.0E-4 |
| GOTERM_CC_FAT | [vesicle](http://www.ebi.ac.uk/QuickGO/GTerm?id=GO:0031982) | 77 | 2.8 | 1.8E-5 | 2.3E-4 |
| GOTERM_MF_FAT | [guanyl nucleotide binding](http://www.ebi.ac.uk/QuickGO/GTerm?id=GO:0019001) | 76 | 2.7 | 7.9E-6 | 2.9E-4 |
| GOTERM_BP_FAT | [coenzyme catabolic process](http://www.ebi.ac.uk/QuickGO/GTerm?id=GO:0009109) | 14 | 0.5 | 5.3E-6 | 3.5E-4 |
| GOTERM_MF_FAT | [ubiquitin-protein ligase activity](http://www.ebi.ac.uk/QuickGO/GTerm?id=GO:0004842) | 23 | 0.8 | 1.1E-5 | 3.8E-4 |
| GOTERM_CC_FAT | [NADH dehydrogenase complex](http://www.ebi.ac.uk/QuickGO/GTerm?id=GO:0030964) | 9 | 0.3 | 3.1E-5 | 4.0E-4 |
| GOTERM_CC_FAT | [mitochondrial respiratory chain complex I](http://www.ebi.ac.uk/QuickGO/GTerm?id=GO:0005747) | 9 | 0.3 | 3.1E-5 | 4.0E-4 |
| GOTERM_CC_FAT | [respiratory chain complex I](http://www.ebi.ac.uk/QuickGO/GTerm?id=GO:0045271) | 9 | 0.3 | 3.1E-5 | 4.0E-4 |
| GOTERM_CC_FAT | [endoplasmic reticulum](http://www.ebi.ac.uk/QuickGO/GTerm?id=GO:0005783) | 112 | 4.0 | 3.4E-5 | 4.3E-4 |
| GOTERM_MF_FAT | [cation-transporting ATPase activity](http://www.ebi.ac.uk/QuickGO/GTerm?id=GO:0019829) | 15 | 0.5 | 1.3E-5 | 4.4E-4 |
| GOTERM_BP_FAT | [nucleobase, nucleoside, nucleotide and nucleic acid transport](http://www.ebi.ac.uk/QuickGO/GTerm?id=GO:0015931) | 18 | 0.6 | 7.0E-6 | 4.5E-4 |
| GOTERM_CC_FAT | [Golgi apparatus](http://www.ebi.ac.uk/QuickGO/GTerm?id=GO:0005794) | 97 | 3.5 | 3.8E-5 | 4.7E-4 |
| GOTERM_BP_FAT | [glucose metabolic process](http://www.ebi.ac.uk/QuickGO/GTerm?id=GO:0006006) | 33 | 1.2 | 7.8E-6 | 4.9E-4 |
| GOTERM_BP_FAT | [regulation of actin filament-based process](http://www.ebi.ac.uk/QuickGO/GTerm?id=GO:0032970) | 17 | 0.6 | 8.1E-6 | 5.0E-4 |
| GOTERM_BP_FAT | [protein ubiquitination](http://www.ebi.ac.uk/QuickGO/GTerm?id=GO:0016567) | 21 | 0.8 | 9.2E-6 | 5.5E-4 |
| GOTERM_CC_FAT | [sarcoplasm](http://www.ebi.ac.uk/QuickGO/GTerm?id=GO:0016528) | 12 | 0.4 | 5.0E-5 | 6.0E-4 |
| GOTERM_BP_FAT | [proteolysis](http://www.ebi.ac.uk/QuickGO/GTerm?id=GO:0006508) | 139 | 5.0 | 1.0E-5 | 6.1E-4 |
| SP_PIR_KEYWORDS | [translation regulation](http://www.uniprot.org/keywords/?query=translation%20regulation) | 15 | 0.5 | 5.0E-5 | 6.3E-4 |
| GOTERM_CC_FAT | [large ribosomal subunit](http://www.ebi.ac.uk/QuickGO/GTerm?id=GO:0015934) | 14 | 0.5 | 5.9E-5 | 6.9E-4 |
| GOTERM_CC_FAT | [cytoplasmic vesicle](http://www.ebi.ac.uk/QuickGO/GTerm?id=GO:0031410) | 73 | 2.6 | 7.0E-5 | 8.0E-4 |
| GOTERM_CC_FAT | [perinuclear region of cytoplasm](http://www.ebi.ac.uk/QuickGO/GTerm?id=GO:0048471) | 36 | 1.3 | 7.9E-5 | 8.9E-4 |
| GOTERM_BP_FAT | [RNA localization](http://www.ebi.ac.uk/QuickGO/GTerm?id=GO:0006403) | 16 | 0.6 | 1.6E-5 | 9.0E-4 |
| GOTERM_BP_FAT | [establishment of RNA localization](http://www.ebi.ac.uk/QuickGO/GTerm?id=GO:0051236) | 16 | 0.6 | 1.6E-5 | 9.0E-4 |
| GOTERM_BP_FAT | [RNA transport](http://www.ebi.ac.uk/QuickGO/GTerm?id=GO:0050658) | 16 | 0.6 | 1.6E-5 | 9.0E-4 |
| GOTERM_BP_FAT | [nucleic acid transport](http://www.ebi.ac.uk/QuickGO/GTerm?id=GO:0050657) | 16 | 0.6 | 1.6E-5 | 9.0E-4 |
| GOTERM_CC_FAT | [extrinsic to membrane](http://www.ebi.ac.uk/QuickGO/GTerm?id=GO:0019898) | 47 | 1.7 | 8.2E-5 | 9.0E-4 |
| SP_PIR_KEYWORDS | [atp-binding](http://www.uniprot.org/keywords/?query=atp-binding) | 153 | 5.5 | 7.7E-5 | 9.3E-4 |
| SP_PIR_KEYWORDS | [muscle protein](http://www.uniprot.org/keywords/?query=muscle%20protein) | 17 | 0.6 | 7.6E-5 | 9.4E-4 |
| GOTERM_BP_FAT | [nucleocytoplasmic transport](http://www.ebi.ac.uk/QuickGO/GTerm?id=GO:0006913) | 23 | 0.8 | 1.7E-5 | 9.5E-4 |
| GOTERM_BP_FAT | [nuclear transport](http://www.ebi.ac.uk/QuickGO/GTerm?id=GO:0051169) | 23 | 0.8 | 1.7E-5 | 9.5E-4 |
| GOTERM_CC_FAT | [nuclear body](http://www.ebi.ac.uk/QuickGO/GTerm?id=GO:0016604) | 25 | 0.9 | 1.0E-4 | 1.1E-3 |
| GOTERM_CC_FAT | [organellar ribosome](http://www.ebi.ac.uk/QuickGO/GTerm?id=GO:0000313) | 13 | 0.5 | 1.0E-4 | 1.1E-3 |
| GOTERM_CC_FAT | [mitochondrial ribosome](http://www.ebi.ac.uk/QuickGO/GTerm?id=GO:0005761) | 13 | 0.5 | 1.0E-4 | 1.1E-3 |
| GOTERM_BP_FAT | [regulation of protein complex assembly](http://www.ebi.ac.uk/QuickGO/GTerm?id=GO:0043254) | 17 | 0.6 | 2.2E-5 | 1.2E-3 |
| GOTERM_MF_FAT | [transcription cofactor activity](http://www.ebi.ac.uk/QuickGO/GTerm?id=GO:0003712) | 27 | 1.0 | 3.7E-5 | 1.2E-3 |
| SP_PIR_KEYWORDS | [tricarboxylic acid cycle](http://www.uniprot.org/keywords/?query=tricarboxylic%20acid%20cycle) | 13 | 0.5 | 1.1E-4 | 1.2E-3 |
| GOTERM_BP_FAT | [ATP metabolic process](http://www.ebi.ac.uk/QuickGO/GTerm?id=GO:0046034) | 31 | 1.1 | 2.3E-5 | 1.3E-3 |
| GOTERM_BP_FAT | [regulation of actin cytoskeleton organization](http://www.ebi.ac.uk/QuickGO/GTerm?id=GO:0032956) | 16 | 0.6 | 2.6E-5 | 1.4E-3 |
| GOTERM_BP_FAT | [regulation of protein polymerization](http://www.ebi.ac.uk/QuickGO/GTerm?id=GO:0032271) | 16 | 0.6 | 2.6E-5 | 1.4E-3 |
| GOTERM_MF_FAT | [NADH dehydrogenase activity](http://www.ebi.ac.uk/QuickGO/GTerm?id=GO:0003954) | 14 | 0.5 | 4.4E-5 | 1.4E-3 |
| GOTERM_BP_FAT | [regulation of translation](http://www.ebi.ac.uk/QuickGO/GTerm?id=GO:0006417) | 22 | 0.8 | 3.1E-5 | 1.6E-3 |
| GOTERM_BP_FAT | [cofactor catabolic process](http://www.ebi.ac.uk/QuickGO/GTerm?id=GO:0051187) | 14 | 0.5 | 3.4E-5 | 1.7E-3 |
| SP_PIR_KEYWORDS | [mitochondrial inner membrane](http://www.uniprot.org/keywords/?query=mitochondrial%20inner%20membrane) | 12 | 0.4 | 1.5E-4 | 1.7E-3 |
| GOTERM_CC_FAT | [cytoskeleton](http://www.ebi.ac.uk/QuickGO/GTerm?id=GO:0005856) | 136 | 4.9 | 1.8E-4 | 1.8E-3 |
| GOTERM_CC_FAT | [proteasome core complex, alpha-subunit complex](http://www.ebi.ac.uk/QuickGO/GTerm?id=GO:0019773) | 7 | 0.3 | 1.8E-4 | 1.9E-3 |
| GOTERM_MF_FAT | [ligase activity, forming aminoacyl-tRNA and related compounds](http://www.ebi.ac.uk/QuickGO/GTerm?id=GO:0016876) | 18 | 0.6 | 6.4E-5 | 1.9E-3 |
| GOTERM_MF_FAT | [aminoacyl-tRNA ligase activity](http://www.ebi.ac.uk/QuickGO/GTerm?id=GO:0004812) | 18 | 0.6 | 6.4E-5 | 1.9E-3 |
| GOTERM_MF_FAT | [ligase activity, forming carbon-oxygen bonds](http://www.ebi.ac.uk/QuickGO/GTerm?id=GO:0016875) | 18 | 0.6 | 6.4E-5 | 1.9E-3 |
| GOTERM_BP_FAT | [protein complex biogenesis](http://www.ebi.ac.uk/QuickGO/GTerm?id=GO:0070271) | 51 | 1.8 | 3.9E-5 | 2.0E-3 |
| GOTERM_BP_FAT | [protein complex assembly](http://www.ebi.ac.uk/QuickGO/GTerm?id=GO:0006461) | 51 | 1.8 | 3.9E-5 | 2.0E-3 |
| GOTERM_BP_FAT | [regulation of cytoskeleton organization](http://www.ebi.ac.uk/QuickGO/GTerm?id=GO:0051493) | 21 | 0.8 | 4.1E-5 | 2.0E-3 |
| GOTERM_BP_FAT | [protein localization in organelle](http://www.ebi.ac.uk/QuickGO/GTerm?id=GO:0033365) | 24 | 0.9 | 4.6E-5 | 2.2E-3 |
| GOTERM_MF_FAT | [nucleoside binding](http://www.ebi.ac.uk/QuickGO/GTerm?id=GO:0001882) | 217 | 7.8 | 8.1E-5 | 2.4E-3 |
| GOTERM_BP_FAT | [purine ribonucleotide metabolic process](http://www.ebi.ac.uk/QuickGO/GTerm?id=GO:0009150) | 36 | 1.3 | 5.1E-5 | 2.4E-3 |
| GOTERM_MF_FAT | [peroxiredoxin activity](http://www.ebi.ac.uk/QuickGO/GTerm?id=GO:0051920) | 7 | 0.3 | 8.8E-5 | 2.5E-3 |
| GOTERM_CC_FAT | [contractile fiber part](http://www.ebi.ac.uk/QuickGO/GTerm?id=GO:0044449) | 19 | 0.7 | 2.6E-4 | 2.6E-3 |
| GOTERM_MF_FAT | [adenyl ribonucleotide binding](http://www.ebi.ac.uk/QuickGO/GTerm?id=GO:0032559) | 204 | 7.4 | 9.7E-5 | 2.7E-3 |
| INTERPRO | [Proteasome, subunit alpha/beta](http://www.ebi.ac.uk/interpro/entry/IPR001353) | 14 | 0.5 | 3.5E-6 | 2.7E-3 |
| GOTERM_BP_FAT | [regulation of actin filament polymerization](http://www.ebi.ac.uk/QuickGO/GTerm?id=GO:0030833) | 14 | 0.5 | 5.8E-5 | 2.7E-3 |
| GOTERM_MF_FAT | [GTPase activity](http://www.ebi.ac.uk/QuickGO/GTerm?id=GO:0003924) | 29 | 1.0 | 1.1E-4 | 2.9E-3 |
| INTERPRO | [RNA recognition motif, RNP-1](http://www.ebi.ac.uk/interpro/entry/IPR000504) | 49 | 1.8 | 2.5E-6 | 3.0E-3 |
| GOTERM_CC_FAT | [heterochromatin](http://www.ebi.ac.uk/QuickGO/GTerm?id=GO:0000792) | 12 | 0.4 | 3.3E-4 | 3.2E-3 |
| SP_PIR_KEYWORDS | [ATP biosynthesis](http://www.uniprot.org/keywords/?query=ATP%20biosynthesis) | 10 | 0.4 | 3.0E-4 | 3.4E-3 |
| INTERPRO | [Nucleotide-binding, alpha-beta plait](http://www.ebi.ac.uk/interpro/entry/IPR012677) | 49 | 1.8 | 1.5E-6 | 3.4E-3 |
| GOTERM_CC_FAT | [membrane-bounded vesicle](http://www.ebi.ac.uk/QuickGO/GTerm?id=GO:0031988) | 61 | 2.2 | 3.7E-4 | 3.5E-3 |
| GOTERM_MF_FAT | [NADH dehydrogenase (quinone) activity](http://www.ebi.ac.uk/QuickGO/GTerm?id=GO:0050136) | 13 | 0.5 | 1.4E-4 | 3.7E-3 |
| GOTERM_MF_FAT | [NADH dehydrogenase (ubiquinone) activity](http://www.ebi.ac.uk/QuickGO/GTerm?id=GO:0008137) | 13 | 0.5 | 1.4E-4 | 3.7E-3 |
| GOTERM_MF_FAT | [ATP binding](http://www.ebi.ac.uk/QuickGO/GTerm?id=GO:0005524) | 201 | 7.2 | 1.6E-4 | 3.9E-3 |
| GOTERM_MF_FAT | [purine nucleoside binding](http://www.ebi.ac.uk/QuickGO/GTerm?id=GO:0001883) | 214 | 7.7 | 1.6E-4 | 4.0E-3 |
| GOTERM_MF_FAT | [adenyl nucleotide binding](http://www.ebi.ac.uk/QuickGO/GTerm?id=GO:0030554) | 212 | 7.6 | 1.7E-4 | 4.1E-3 |
| KEGG_PATHWAY | [Aminoacyl-tRNA biosynthesis](http://david.abcc.ncifcrf.gov/kegg.jsp?path=bta00970$Aminoacyl-tRNA%20biosynthesis&termId=470015018&source=kegg) | 18 | 0.6 | 2.5E-4 | 4.2E-3 |
| GOTERM_BP_FAT | [mRNA transport](http://www.ebi.ac.uk/QuickGO/GTerm?id=GO:0051028) | 14 | 0.5 | 9.6E-5 | 4.4E-3 |
| GOTERM_BP_FAT | [regulation of actin filament length](http://www.ebi.ac.uk/QuickGO/GTerm?id=GO:0030832) | 14 | 0.5 | 9.6E-5 | 4.4E-3 |
| GOTERM_BP_FAT | [regulation of actin polymerization or depolymerization](http://www.ebi.ac.uk/QuickGO/GTerm?id=GO:0008064) | 14 | 0.5 | 9.6E-5 | 4.4E-3 |
| GOTERM_BP_FAT | [cellular homeostasis](http://www.ebi.ac.uk/QuickGO/GTerm?id=GO:0019725) | 57 | 2.1 | 9.9E-5 | 4.4E-3 |
| GOTERM_BP_FAT | [mitochondrion organization](http://www.ebi.ac.uk/QuickGO/GTerm?id=GO:0007005) | 25 | 0.9 | 1.0E-4 | 4.6E-3 |
| SMART | [UBCc](http://smart.embl.de/smart/do_annotation.pl?DOMAIN=SM00212) | 17 | 0.6 | 5.0E-5 | 4.9E-3 |
| GOTERM_CC_FAT | [nuclear speck](http://www.ebi.ac.uk/QuickGO/GTerm?id=GO:0016607) | 17 | 0.6 | 5.5E-4 | 5.0E-3 |
| GOTERM_CC_FAT | [organellar large ribosomal subunit](http://www.ebi.ac.uk/QuickGO/GTerm?id=GO:0000315) | 9 | 0.3 | 5.3E-4 | 5.0E-3 |
| GOTERM_CC_FAT | [mitochondrial large ribosomal subunit](http://www.ebi.ac.uk/QuickGO/GTerm?id=GO:0005762) | 9 | 0.3 | 5.3E-4 | 5.0E-3 |
| GOTERM_CC_FAT | [coated membrane](http://www.ebi.ac.uk/QuickGO/GTerm?id=GO:0048475) | 20 | 0.7 | 5.5E-4 | 5.1E-3 |
| GOTERM_CC_FAT | [membrane coat](http://www.ebi.ac.uk/QuickGO/GTerm?id=GO:0030117) | 20 | 0.7 | 5.5E-4 | 5.1E-3 |
| GOTERM_CC_FAT | [sarcoplasmic reticulum](http://www.ebi.ac.uk/QuickGO/GTerm?id=GO:0016529) | 10 | 0.4 | 5.9E-4 | 5.3E-3 |
| GOTERM_MF_FAT | [purine NTP-dependent helicase activity](http://www.ebi.ac.uk/QuickGO/GTerm?id=GO:0070035) | 22 | 0.8 | 2.3E-4 | 5.3E-3 |
| GOTERM_MF_FAT | [ATP-dependent helicase activity](http://www.ebi.ac.uk/QuickGO/GTerm?id=GO:0008026) | 22 | 0.8 | 2.3E-4 | 5.3E-3 |
| GOTERM_MF_FAT | [ATPase activity, coupled](http://www.ebi.ac.uk/QuickGO/GTerm?id=GO:0042623) | 47 | 1.7 | 2.4E-4 | 5.4E-3 |
| SP_PIR_KEYWORDS | [mRNA transport](http://www.uniprot.org/keywords/?query=mRNA%20transport) | 15 | 0.5 | 5.0E-4 | 5.5E-3 |
| SMART | [PINT](http://smart.embl.de/smart/do_annotation.pl?DOMAIN=SM00088) | 11 | 0.4 | 7.7E-5 | 5.7E-3 |
| GOTERM_BP_FAT | [ribonucleotide metabolic process](http://www.ebi.ac.uk/QuickGO/GTerm?id=GO:0009259) | 36 | 1.3 | 1.4E-4 | 5.9E-3 |
| SP_PIR_KEYWORDS | [gtp-binding](http://www.uniprot.org/keywords/?query=gtp-binding) | 66 | 2.4 | 6.1E-4 | 6.5E-3 |
| GOTERM_CC_FAT | [sarcomere](http://www.ebi.ac.uk/QuickGO/GTerm?id=GO:0030017) | 17 | 0.6 | 7.7E-4 | 6.8E-3 |
| KEGG_PATHWAY | [Cardiac muscle contraction](http://david.abcc.ncifcrf.gov/kegg.jsp?path=bta04260$Cardiac%20muscle%20contraction&termId=470015055&source=kegg) | 25 | 0.9 | 4.5E-4 | 6.8E-3 |
| GOTERM_BP_FAT | [amino acid activation](http://www.ebi.ac.uk/QuickGO/GTerm?id=GO:0043038) | 17 | 0.6 | 1.7E-4 | 7.1E-3 |
| GOTERM_BP_FAT | [tRNA aminoacylation for protein translation](http://www.ebi.ac.uk/QuickGO/GTerm?id=GO:0006418) | 17 | 0.6 | 1.7E-4 | 7.1E-3 |
| GOTERM_BP_FAT | [tRNA aminoacylation](http://www.ebi.ac.uk/QuickGO/GTerm?id=GO:0043039) | 17 | 0.6 | 1.7E-4 | 7.1E-3 |
| KEGG_PATHWAY | [Tight junction](http://david.abcc.ncifcrf.gov/kegg.jsp?path=bta04530$Tight%20junction&termId=470015068&source=kegg) | 38 | 1.4 | 5.1E-4 | 7.1E-3 |
| GOTERM_BP_FAT | [purine nucleotide metabolic process](http://www.ebi.ac.uk/QuickGO/GTerm?id=GO:0006163) | 39 | 1.4 | 1.7E-4 | 7.2E-3 |
| SMART | [UBQ](http://smart.embl.de/smart/do_annotation.pl?DOMAIN=SM00213) | 18 | 0.6 | 4.9E-5 | 7.3E-3 |
| GOTERM_BP_FAT | [purine ribonucleoside triphosphate metabolic process](http://www.ebi.ac.uk/QuickGO/GTerm?id=GO:0009205) | 31 | 1.1 | 1.8E-4 | 7.4E-3 |
| GOTERM_BP_FAT | [ribonucleoside triphosphate metabolic process](http://www.ebi.ac.uk/QuickGO/GTerm?id=GO:0009199) | 31 | 1.1 | 1.8E-4 | 7.4E-3 |
| SMART | [UBA](http://smart.embl.de/smart/do_annotation.pl?DOMAIN=SM00165) | 13 | 0.5 | 1.3E-4 | 7.4E-3 |
| GOTERM_CC_FAT | [Z disc](http://www.ebi.ac.uk/QuickGO/GTerm?id=GO:0030018) | 12 | 0.4 | 8.9E-4 | 7.7E-3 |
| GOTERM_CC_FAT | [nuclear chromatin](http://www.ebi.ac.uk/QuickGO/GTerm?id=GO:0000790) | 12 | 0.4 | 8.9E-4 | 7.7E-3 |
| GOTERM_MF_FAT | [enzyme binding](http://www.ebi.ac.uk/QuickGO/GTerm?id=GO:0019899) | 45 | 1.6 | 3.5E-4 | 7.8E-3 |
| SP_PIR_KEYWORDS | [ligase](http://www.uniprot.org/keywords/?query=ligase) | 40 | 1.4 | 7.9E-4 | 8.2E-3 |
| GOTERM_BP_FAT | [regulation of organelle organization](http://www.ebi.ac.uk/QuickGO/GTerm?id=GO:0033043) | 26 | 0.9 | 2.1E-4 | 8.2E-3 |
| GOTERM_BP_FAT | [ncRNA metabolic process](http://www.ebi.ac.uk/QuickGO/GTerm?id=GO:0034660) | 39 | 1.4 | 2.0E-4 | 8.3E-3 |
| GOTERM_BP_FAT | [intra-Golgi vesicle-mediated transport](http://www.ebi.ac.uk/QuickGO/GTerm?id=GO:0006891) | 8 | 0.3 | 2.1E-4 | 8.3E-3 |
| GOTERM_CC_FAT | [proton-transporting two-sector ATPase complex, catalytic domain](http://www.ebi.ac.uk/QuickGO/GTerm?id=GO:0033178) | 11 | 0.4 | 9.9E-4 | 8.4E-3 |
| SMART | [PP2Ac](http://smart.embl.de/smart/do_annotation.pl?DOMAIN=SM00156) | 9 | 0.3 | 1.7E-4 | 8.5E-3 |
| GOTERM_BP_FAT | [purine nucleoside triphosphate metabolic process](http://www.ebi.ac.uk/QuickGO/GTerm?id=GO:0009144) | 31 | 1.1 | 2.2E-4 | 8.5E-3 |
| GOTERM_CC_FAT | [endosome](http://www.ebi.ac.uk/QuickGO/GTerm?id=GO:0005768) | 35 | 1.3 | 1.0E-3 | 8.5E-3 |
| KEGG_PATHWAY | [Protein export](http://david.abcc.ncifcrf.gov/kegg.jsp?path=bta03060$Protein%20export&termId=470015031&source=kegg) | 7 | 0.3 | 6.6E-4 | 8.5E-3 |
| GOTERM_MF_FAT | [mRNA binding](http://www.ebi.ac.uk/QuickGO/GTerm?id=GO:0003729) | 10 | 0.4 | 4.0E-4 | 8.7E-3 |
| GOTERM_CC_FAT | [ER-Golgi intermediate compartment](http://www.ebi.ac.uk/QuickGO/GTerm?id=GO:0005793) | 10 | 0.4 | 1.1E-3 | 8.7E-3 |
| GOTERM_BP_FAT | [ribonucleoprotein complex biogenesis](http://www.ebi.ac.uk/QuickGO/GTerm?id=GO:0022613) | 28 | 1.0 | 2.3E-4 | 8.7E-3 |
| GOTERM_BP_FAT | [cellular protein complex assembly](http://www.ebi.ac.uk/QuickGO/GTerm?id=GO:0043623) | 28 | 1.0 | 2.3E-4 | 8.7E-3 |
| GOTERM_CC_FAT | [cytoplasmic membrane-bounded vesicle](http://www.ebi.ac.uk/QuickGO/GTerm?id=GO:0016023) | 58 | 2.1 | 1.1E-3 | 8.8E-3 |
| GOTERM_MF_FAT | [hydrogen ion transporting ATP synthase activity, rotational mechanism](http://www.ebi.ac.uk/QuickGO/GTerm?id=GO:0046933) | 9 | 0.3 | 4.3E-4 | 9.2E-3 |
| GOTERM_BP_FAT | [ATP biosynthetic process](http://www.ebi.ac.uk/QuickGO/GTerm?id=GO:0006754) | 27 | 1.0 | 2.4E-4 | 9.2E-3 |
| GOTERM_CC_FAT | [nucleolus](http://www.ebi.ac.uk/QuickGO/GTerm?id=GO:0005730) | 48 | 1.7 | 1.2E-3 | 9.8E-3 |
| SP_PIR_KEYWORDS | [atp synthesis](http://www.uniprot.org/keywords/?query=atp%20synthesis) | 9 | 0.3 | 9.8E-4 | 1.0E-2 |
| GOTERM_BP_FAT | [Golgi vesicle transport](http://www.ebi.ac.uk/QuickGO/GTerm?id=GO:0048193) | 15 | 0.5 | 2.8E-4 | 1.0E-2 |
| GOTERM_CC_FAT | [proton-transporting two-sector ATPase complex, proton-transporting domain](http://www.ebi.ac.uk/QuickGO/GTerm?id=GO:0033177) | 12 | 0.4 | 1.4E-3 | 1.1E-2 |
| GOTERM_MF_FAT | [oxidoreductase activity, acting on NADH or NADPH, quinone or similar compound as acceptor](http://www.ebi.ac.uk/QuickGO/GTerm?id=GO:0016655) | 13 | 0.5 | 5.4E-4 | 1.1E-2 |
| COG_ONTOLOGY | [Translation, ribosomal structure and biogenesis](http://www.ncbi.nlm.nih.gov/COG/new/) | 19 | 0.7 | 6.5E-4 | 1.1E-2 |
| INTERPRO | [Nucleic acid-binding, OB-fold](http://www.ebi.ac.uk/interpro/entry/IPR012340) | 22 | 0.8 | 2.1E-5 | 1.2E-2 |
| GOTERM_CC_FAT | [COPI coated vesicle membrane](http://www.ebi.ac.uk/QuickGO/GTerm?id=GO:0030663) | 7 | 0.3 | 1.6E-3 | 1.2E-2 |
| GOTERM_CC_FAT | [COPI vesicle coat](http://www.ebi.ac.uk/QuickGO/GTerm?id=GO:0030126) | 7 | 0.3 | 1.6E-3 | 1.2E-2 |
| GOTERM_CC_FAT | [ubiquitin ligase complex](http://www.ebi.ac.uk/QuickGO/GTerm?id=GO:0000151) | 15 | 0.5 | 1.6E-3 | 1.2E-2 |
| GOTERM_MF_FAT | [ATPase activity](http://www.ebi.ac.uk/QuickGO/GTerm?id=GO:0016887) | 53 | 1.9 | 6.4E-4 | 1.3E-2 |
| SP_PIR_KEYWORDS | [cytoplasmic vesicle](http://www.uniprot.org/keywords/?query=cytoplasmic%20vesicle) | 36 | 1.3 | 1.3E-3 | 1.3E-2 |
| GOTERM_CC_FAT | [mitochondrial outer membrane](http://www.ebi.ac.uk/QuickGO/GTerm?id=GO:0005741) | 16 | 0.6 | 1.8E-3 | 1.3E-2 |
| SP_PIR_KEYWORDS | [endosome](http://www.uniprot.org/keywords/?query=endosome) | 26 | 0.9 | 1.4E-3 | 1.3E-2 |
| GOTERM_MF_FAT | [proton-transporting ATPase activity, rotational mechanism](http://www.ebi.ac.uk/QuickGO/GTerm?id=GO:0046961) | 10 | 0.4 | 6.9E-4 | 1.4E-2 |
| GOTERM_BP_FAT | [ribosome biogenesis](http://www.ebi.ac.uk/QuickGO/GTerm?id=GO:0042254) | 24 | 0.9 | 3.8E-4 | 1.4E-2 |
| INTERPRO | [Ubiquitin-conjugating enzyme/RWD-like](http://www.ebi.ac.uk/interpro/entry/IPR016135) | 19 | 0.7 | 3.0E-5 | 1.4E-2 |
| GOTERM_CC_FAT | [organelle outer membrane](http://www.ebi.ac.uk/QuickGO/GTerm?id=GO:0031968) | 17 | 0.6 | 1.9E-3 | 1.4E-2 |
| GOTERM_CC_FAT | [nuclear heterochromatin](http://www.ebi.ac.uk/QuickGO/GTerm?id=GO:0005720) | 9 | 0.3 | 1.9E-3 | 1.4E-2 |
| GOTERM_CC_FAT | [proton-transporting ATP synthase complex, coupling factor F(o)](http://www.ebi.ac.uk/QuickGO/GTerm?id=GO:0045263) | 9 | 0.3 | 1.9E-3 | 1.4E-2 |
| GOTERM_BP_FAT | [regulation of cellular component size](http://www.ebi.ac.uk/QuickGO/GTerm?id=GO:0032535) | 26 | 0.9 | 4.1E-4 | 1.5E-2 |
| GOTERM_CC_FAT | [I band](http://www.ebi.ac.uk/QuickGO/GTerm?id=GO:0031674) | 12 | 0.4 | 2.1E-3 | 1.5E-2 |
| GOTERM_BP_FAT | [macromolecular complex subunit organization](http://www.ebi.ac.uk/QuickGO/GTerm?id=GO:0043933) | 73 | 2.6 | 4.2E-4 | 1.5E-2 |
| GOTERM_BP_FAT | [proteasomal protein catabolic process](http://www.ebi.ac.uk/QuickGO/GTerm?id=GO:0010498) | 11 | 0.4 | 4.3E-4 | 1.5E-2 |
| GOTERM_BP_FAT | [proteasomal ubiquitin-dependent protein catabolic process](http://www.ebi.ac.uk/QuickGO/GTerm?id=GO:0043161) | 11 | 0.4 | 4.3E-4 | 1.5E-2 |
| GOTERM_BP_FAT | [glycolysis](http://www.ebi.ac.uk/QuickGO/GTerm?id=GO:0006096) | 16 | 0.6 | 4.4E-4 | 1.5E-2 |
| GOTERM_BP_FAT | [purine ribonucleotide biosynthetic process](http://www.ebi.ac.uk/QuickGO/GTerm?id=GO:0009152) | 31 | 1.1 | 4.7E-4 | 1.6E-2 |
| GOTERM_BP_FAT | [glucose catabolic process](http://www.ebi.ac.uk/QuickGO/GTerm?id=GO:0006007) | 18 | 0.6 | 4.6E-4 | 1.6E-2 |
| GOTERM_BP_FAT | [ATP synthesis coupled electron transport](http://www.ebi.ac.uk/QuickGO/GTerm?id=GO:0042773) | 10 | 0.4 | 4.9E-4 | 1.6E-2 |
| GOTERM_MF_FAT | [transcription coactivator activity](http://www.ebi.ac.uk/QuickGO/GTerm?id=GO:0003713) | 16 | 0.6 | 8.5E-4 | 1.6E-2 |
| GOTERM_CC_FAT | [microtubule cytoskeleton](http://www.ebi.ac.uk/QuickGO/GTerm?id=GO:0015630) | 57 | 2.1 | 2.3E-3 | 1.6E-2 |
| GOTERM_BP_FAT | [nucleoside triphosphate metabolic process](http://www.ebi.ac.uk/QuickGO/GTerm?id=GO:0009141) | 32 | 1.2 | 5.1E-4 | 1.7E-2 |
| GOTERM_BP_FAT | [cellular macromolecular complex subunit organization](http://www.ebi.ac.uk/QuickGO/GTerm?id=GO:0034621) | 52 | 1.9 | 5.1E-4 | 1.7E-2 |
| GOTERM_MF_FAT | [helicase activity](http://www.ebi.ac.uk/QuickGO/GTerm?id=GO:0004386) | 28 | 1.0 | 8.8E-4 | 1.7E-2 |
| GOTERM_BP_FAT | [mitochondrial ATP synthesis coupled electron transport](http://www.ebi.ac.uk/QuickGO/GTerm?id=GO:0042775) | 9 | 0.3 | 5.2E-4 | 1.7E-2 |
| GOTERM_CC_FAT | [pore complex](http://www.ebi.ac.uk/QuickGO/GTerm?id=GO:0046930) | 16 | 0.6 | 2.4E-3 | 1.7E-2 |
| GOTERM_BP_FAT | [hexose metabolic process](http://www.ebi.ac.uk/QuickGO/GTerm?id=GO:0019318) | 33 | 1.2 | 5.4E-4 | 1.7E-2 |
| KEGG_PATHWAY | [Renal cell carcinoma](http://david.abcc.ncifcrf.gov/kegg.jsp?path=bta05211$Renal%20cell%20carcinoma&termId=470015109&source=kegg) | 23 | 0.8 | 1.4E-3 | 1.7E-2 |
| SP_PIR_KEYWORDS | [electron transfer](http://www.uniprot.org/keywords/?query=electron%20transfer) | 14 | 0.5 | 1.9E-3 | 1.8E-2 |
| SP_PIR_KEYWORDS | [Aminoacyl-tRNA synthetase](http://www.uniprot.org/keywords/?query=Aminoacyl-tRNA%20synthetase) | 14 | 0.5 | 1.9E-3 | 1.8E-2 |
| GOTERM_CC_FAT | [NuRD complex](http://www.ebi.ac.uk/QuickGO/GTerm?id=GO:0016581) | 6 | 0.2 | 2.7E-3 | 1.9E-2 |
| GOTERM_CC_FAT | [integral to mitochondrial membrane](http://www.ebi.ac.uk/QuickGO/GTerm?id=GO:0032592) | 6 | 0.2 | 2.7E-3 | 1.9E-2 |
| GOTERM_BP_FAT | [cellular carbohydrate catabolic process](http://www.ebi.ac.uk/QuickGO/GTerm?id=GO:0044275) | 20 | 0.7 | 5.9E-4 | 1.9E-2 |
| GOTERM_CC_FAT | [actin cytoskeleton](http://www.ebi.ac.uk/QuickGO/GTerm?id=GO:0015629) | 32 | 1.2 | 2.8E-3 | 1.9E-2 |
| KEGG_PATHWAY | [Insulin signaling pathway](http://david.abcc.ncifcrf.gov/kegg.jsp?path=bta04910$Insulin%20signaling%20pathway&termId=470015093&source=kegg) | 37 | 1.3 | 1.7E-3 | 1.9E-2 |
| GOTERM_BP_FAT | [hexose catabolic process](http://www.ebi.ac.uk/QuickGO/GTerm?id=GO:0019320) | 18 | 0.6 | 6.2E-4 | 1.9E-2 |
| GOTERM_CC_FAT | [sarcolemma](http://www.ebi.ac.uk/QuickGO/GTerm?id=GO:0042383) | 12 | 0.4 | 3.0E-3 | 2.0E-2 |
| GOTERM_CC_FAT | [outer membrane](http://www.ebi.ac.uk/QuickGO/GTerm?id=GO:0019867) | 17 | 0.6 | 3.3E-3 | 2.2E-2 |
| GOTERM_CC_FAT | [nuclear envelope](http://www.ebi.ac.uk/QuickGO/GTerm?id=GO:0005635) | 28 | 1.0 | 3.5E-3 | 2.2E-2 |
| GOTERM_CC_FAT | [nuclear DNA-directed RNA polymerase complex](http://www.ebi.ac.uk/QuickGO/GTerm?id=GO:0055029) | 8 | 0.3 | 3.5E-3 | 2.2E-2 |
| GOTERM_CC_FAT | [RNA polymerase complex](http://www.ebi.ac.uk/QuickGO/GTerm?id=GO:0030880) | 8 | 0.3 | 3.5E-3 | 2.2E-2 |
| GOTERM_CC_FAT | [DNA-directed RNA polymerase complex](http://www.ebi.ac.uk/QuickGO/GTerm?id=GO:0000428) | 8 | 0.3 | 3.5E-3 | 2.2E-2 |
| GOTERM_CC_FAT | [COPI-coated vesicle](http://www.ebi.ac.uk/QuickGO/GTerm?id=GO:0030137) | 7 | 0.3 | 3.4E-3 | 2.2E-2 |
| GOTERM_CC_FAT | [prefoldin complex](http://www.ebi.ac.uk/QuickGO/GTerm?id=GO:0016272) | 7 | 0.3 | 3.4E-3 | 2.2E-2 |
| GOTERM_CC_FAT | [lamellipodium](http://www.ebi.ac.uk/QuickGO/GTerm?id=GO:0030027) | 13 | 0.5 | 3.5E-3 | 2.2E-2 |
| KEGG_PATHWAY | [Glycolysis / Gluconeogenesis](http://david.abcc.ncifcrf.gov/kegg.jsp?path=bta00010$Glycolysis%20/%20Gluconeogenesis&termId=470014937&source=kegg) | 20 | 0.7 | 2.3E-3 | 2.4E-2 |
| GOTERM_BP_FAT | [monosaccharide catabolic process](http://www.ebi.ac.uk/QuickGO/GTerm?id=GO:0046365) | 18 | 0.6 | 8.2E-4 | 2.5E-2 |
| GOTERM_BP_FAT | [nuclear-transcribed mRNA catabolic process](http://www.ebi.ac.uk/QuickGO/GTerm?id=GO:0000956) | 10 | 0.4 | 8.4E-4 | 2.5E-2 |
| SP_PIR_KEYWORDS | [tpr repeat](http://www.uniprot.org/keywords/?query=tpr%20repeat) | 22 | 0.8 | 2.8E-3 | 2.6E-2 |
| GOTERM_CC_FAT | [chaperonin-containing T-complex](http://www.ebi.ac.uk/QuickGO/GTerm?id=GO:0005832) | 5 | 0.2 | 4.3E-3 | 2.6E-2 |
| GOTERM_MF_FAT | [oligosaccharyl transferase activity](http://www.ebi.ac.uk/QuickGO/GTerm?id=GO:0004576) | 6 | 0.2 | 1.5E-3 | 2.7E-2 |
| GOTERM_BP_FAT | [retrograde vesicle-mediated transport, Golgi to ER](http://www.ebi.ac.uk/QuickGO/GTerm?id=GO:0006890) | 7 | 0.3 | 9.1E-4 | 2.7E-2 |
| GOTERM_BP_FAT | [negative regulation of protein complex assembly](http://www.ebi.ac.uk/QuickGO/GTerm?id=GO:0031333) | 8 | 0.3 | 1.0E-3 | 3.0E-2 |
| GOTERM_BP_FAT | [negative regulation of protein polymerization](http://www.ebi.ac.uk/QuickGO/GTerm?id=GO:0032272) | 8 | 0.3 | 1.0E-3 | 3.0E-2 |
| INTERPRO | [Ubiquitin supergroup](http://www.ebi.ac.uk/interpro/entry/IPR019955) | 19 | 0.7 | 8.1E-5 | 3.1E-2 |
| GOTERM_MF_FAT | [translation elongation factor activity](http://www.ebi.ac.uk/QuickGO/GTerm?id=GO:0003746) | 10 | 0.4 | 1.8E-3 | 3.2E-2 |
| GOTERM_BP_FAT | [actin cytoskeleton organization](http://www.ebi.ac.uk/QuickGO/GTerm?id=GO:0030036) | 26 | 0.9 | 1.1E-3 | 3.2E-2 |
| GOTERM_BP_FAT | [cell cycle](http://www.ebi.ac.uk/QuickGO/GTerm?id=GO:0007049) | 62 | 2.2 | 1.1E-3 | 3.2E-2 |
| GOTERM_BP_FAT | [ribonucleotide biosynthetic process](http://www.ebi.ac.uk/QuickGO/GTerm?id=GO:0009260) | 31 | 1.1 | 1.1E-3 | 3.3E-2 |
| GOTERM_BP_FAT | [alcohol catabolic process](http://www.ebi.ac.uk/QuickGO/GTerm?id=GO:0046164) | 20 | 0.7 | 1.2E-3 | 3.4E-2 |
| SMART | [DEXDc](http://smart.embl.de/smart/do_annotation.pl?DOMAIN=SM00487) | 20 | 0.7 | 8.2E-4 | 3.4E-2 |
| GOTERM_BP_FAT | [purine nucleotide biosynthetic process](http://www.ebi.ac.uk/QuickGO/GTerm?id=GO:0006164) | 33 | 1.2 | 1.2E-3 | 3.4E-2 |
| GOTERM_BP_FAT | [purine ribonucleoside triphosphate biosynthetic process](http://www.ebi.ac.uk/QuickGO/GTerm?id=GO:0009206) | 27 | 1.0 | 1.2E-3 | 3.4E-2 |
| GOTERM_BP_FAT | [purine nucleoside triphosphate biosynthetic process](http://www.ebi.ac.uk/QuickGO/GTerm?id=GO:0009145) | 27 | 1.0 | 1.2E-3 | 3.4E-2 |
| GOTERM_BP_FAT | [ribonucleoside triphosphate biosynthetic process](http://www.ebi.ac.uk/QuickGO/GTerm?id=GO:0009201) | 27 | 1.0 | 1.2E-3 | 3.4E-2 |
| INTERPRO | [Ubiquitin](http://www.ebi.ac.uk/interpro/entry/IPR000626) | 18 | 0.6 | 1.3E-4 | 3.6E-2 |
| INTERPRO | [Proteasome component region PCI](http://www.ebi.ac.uk/interpro/entry/IPR000717) | 11 | 0.4 | 1.4E-4 | 3.7E-2 |
| GOTERM_BP_FAT | [mRNA catabolic process](http://www.ebi.ac.uk/QuickGO/GTerm?id=GO:0006402) | 10 | 0.4 | 1.4E-3 | 3.7E-2 |
| GOTERM_CC_FAT | [proton-transporting ATP synthase complex, catalytic core F(1)](http://www.ebi.ac.uk/QuickGO/GTerm?id=GO:0045261) | 6 | 0.2 | 6.1E-3 | 3.7E-2 |
| GOTERM_CC_FAT | [nuclear pore](http://www.ebi.ac.uk/QuickGO/GTerm?id=GO:0005643) | 13 | 0.5 | 6.4E-3 | 3.8E-2 |
| SP_PIR_KEYWORDS | [glycolysis](http://www.uniprot.org/keywords/?query=glycolysis) | 14 | 0.5 | 4.2E-3 | 3.8E-2 |
| GOTERM_CC_FAT | [mitochondrial proton-transporting ATP synthase complex](http://www.ebi.ac.uk/QuickGO/GTerm?id=GO:0005753) | 7 | 0.3 | 6.3E-3 | 3.8E-2 |
| GOTERM_BP_FAT | [nucleoside triphosphate biosynthetic process](http://www.ebi.ac.uk/QuickGO/GTerm?id=GO:0009142) | 27 | 1.0 | 1.5E-3 | 3.9E-2 |
| GOTERM_CC_FAT | [nuclear chromosome part](http://www.ebi.ac.uk/QuickGO/GTerm?id=GO:0044454) | 16 | 0.6 | 6.8E-3 | 4.0E-2 |
| GOTERM_CC_FAT | [cell leading edge](http://www.ebi.ac.uk/QuickGO/GTerm?id=GO:0031252) | 17 | 0.6 | 6.7E-3 | 4.0E-2 |
| GOTERM_BP_FAT | [cytoskeleton organization](http://www.ebi.ac.uk/QuickGO/GTerm?id=GO:0007010) | 43 | 1.6 | 1.5E-3 | 4.0E-2 |
| SMART | [HELICc](http://smart.embl.de/smart/do_annotation.pl?DOMAIN=SM00490) | 20 | 0.7 | 1.1E-3 | 4.0E-2 |
| INTERPRO | [Ubiquitin-conjugating enzyme, E2](http://www.ebi.ac.uk/interpro/entry/IPR000608) | 17 | 0.6 | 1.2E-4 | 4.1E-2 |
| SMART | [KOW](http://smart.embl.de/smart/do_annotation.pl?DOMAIN=SM00739) | 6 | 0.2 | 1.3E-3 | 4.2E-2 |
| GOTERM_BP_FAT | [muscle tissue development](http://www.ebi.ac.uk/QuickGO/GTerm?id=GO:0060537) | 22 | 0.8 | 1.6E-3 | 4.3E-2 |
| GOTERM_BP_FAT | [actin filament-based process](http://www.ebi.ac.uk/QuickGO/GTerm?id=GO:0030029) | 26 | 0.9 | 1.6E-3 | 4.3E-2 |
| GOTERM_CC_FAT | [cell fraction](http://www.ebi.ac.uk/QuickGO/GTerm?id=GO:0000267) | 58 | 2.1 | 7.9E-3 | 4.5E-2 |
| KEGG_PATHWAY | [RNA polymerase](http://david.abcc.ncifcrf.gov/kegg.jsp?path=bta03020$RNA%20polymerase&termId=470015026&source=kegg) | 12 | 0.4 | 4.6E-3 | 4.5E-2 |
| GOTERM_CC_FAT | [proton-transporting V-type ATPase complex](http://www.ebi.ac.uk/QuickGO/GTerm?id=GO:0033176) | 9 | 0.3 | 7.9E-3 | 4.5E-2 |
| GOTERM_BP_FAT | [regulation of binding](http://www.ebi.ac.uk/QuickGO/GTerm?id=GO:0051098) | 18 | 0.6 | 1.8E-3 | 4.6E-2 |
| GOTERM_CC_FAT | [Golgi apparatus part](http://www.ebi.ac.uk/QuickGO/GTerm?id=GO:0044431) | 35 | 1.3 | 9.0E-3 | 5.1E-2 |
| GOTERM_CC_FAT | [nucleoid](http://www.ebi.ac.uk/QuickGO/GTerm?id=GO:0009295) | 8 | 0.3 | 9.4E-3 | 5.2E-2 |
| GOTERM_CC_FAT | [mitochondrial nucleoid](http://www.ebi.ac.uk/QuickGO/GTerm?id=GO:0042645) | 8 | 0.3 | 9.4E-3 | 5.2E-2 |
| GOTERM_CC_FAT | [transcriptional repressor complex](http://www.ebi.ac.uk/QuickGO/GTerm?id=GO:0017053) | 8 | 0.3 | 9.4E-3 | 5.2E-2 |
| SMART | [Sm](http://smart.embl.de/smart/do_annotation.pl?DOMAIN=SM00651) | 9 | 0.3 | 1.8E-3 | 5.2E-2 |
| SP_PIR_KEYWORDS | [elongation factor](http://www.uniprot.org/keywords/?query=elongation%20factor) | 8 | 0.3 | 6.0E-3 | 5.3E-2 |
| SP_PIR_KEYWORDS | [cf(0)](http://www.uniprot.org/keywords/?query=cf(0)) | 8 | 0.3 | 6.0E-3 | 5.3E-2 |
| GOTERM_BP_FAT | [regulation of cellular protein metabolic process](http://www.ebi.ac.uk/QuickGO/GTerm?id=GO:0032268) | 42 | 1.5 | 2.1E-3 | 5.4E-2 |
| GOTERM_BP_FAT | [RNA catabolic process](http://www.ebi.ac.uk/QuickGO/GTerm?id=GO:0006401) | 10 | 0.4 | 2.1E-3 | 5.4E-2 |
| GOTERM_MF_FAT | [disulfide oxidoreductase activity](http://www.ebi.ac.uk/QuickGO/GTerm?id=GO:0015036) | 7 | 0.3 | 3.3E-3 | 5.7E-2 |
| GOTERM_MF_FAT | [metal cluster binding](http://www.ebi.ac.uk/QuickGO/GTerm?id=GO:0051540) | 15 | 0.5 | 3.5E-3 | 5.8E-2 |
| GOTERM_MF_FAT | [iron-sulfur cluster binding](http://www.ebi.ac.uk/QuickGO/GTerm?id=GO:0051536) | 15 | 0.5 | 3.5E-3 | 5.8E-2 |
| INTERPRO | [Ubiquitin-associated/translation elongation factor EF1B, N-terminal, eukaryote](http://www.ebi.ac.uk/interpro/entry/IPR015940) | 13 | 0.5 | 2.6E-4 | 5.8E-2 |
| GOTERM_MF_FAT | [protein disulfide oxidoreductase activity](http://www.ebi.ac.uk/QuickGO/GTerm?id=GO:0015035) | 6 | 0.2 | 3.4E-3 | 5.9E-2 |
| SP_PIR_KEYWORDS | [Redox-active center](http://www.uniprot.org/keywords/?query=Redox-active%20center) | 12 | 0.4 | 6.8E-3 | 5.9E-2 |
| INTERPRO | [Serine/threonine-specific protein phosphatase and bis(5-nucleosyl)-tetraphosphatase](http://www.ebi.ac.uk/interpro/entry/IPR006186) | 9 | 0.3 | 2.9E-4 | 6.0E-2 |
| GOTERM_CC_FAT | [insoluble fraction](http://www.ebi.ac.uk/QuickGO/GTerm?id=GO:0005626) | 52 | 1.9 | 1.1E-2 | 6.3E-2 |
| GOTERM_CC_FAT | [DNA-directed RNA polymerase II, core complex](http://www.ebi.ac.uk/QuickGO/GTerm?id=GO:0005665) | 6 | 0.2 | 1.2E-2 | 6.4E-2 |
| GOTERM_MF_FAT | [heme-copper terminal oxidase activity](http://www.ebi.ac.uk/QuickGO/GTerm?id=GO:0015002) | 10 | 0.4 | 3.9E-3 | 6.4E-2 |
| GOTERM_MF_FAT | [cytochrome-c oxidase activity](http://www.ebi.ac.uk/QuickGO/GTerm?id=GO:0004129) | 10 | 0.4 | 3.9E-3 | 6.4E-2 |
| GOTERM_MF_FAT | [oxidoreductase activity, acting on heme group of donors, oxygen as acceptor](http://www.ebi.ac.uk/QuickGO/GTerm?id=GO:0016676) | 10 | 0.4 | 3.9E-3 | 6.4E-2 |
| GOTERM_MF_FAT | [oxidoreductase activity, acting on heme group of donors](http://www.ebi.ac.uk/QuickGO/GTerm?id=GO:0016675) | 10 | 0.4 | 3.9E-3 | 6.4E-2 |
| GOTERM_MF_FAT | [oxidoreductase activity, acting on sulfur group of donors](http://www.ebi.ac.uk/QuickGO/GTerm?id=GO:0016667) | 11 | 0.4 | 4.1E-3 | 6.6E-2 |
| GOTERM_BP_FAT | [macromolecular complex assembly](http://www.ebi.ac.uk/QuickGO/GTerm?id=GO:0065003) | 66 | 2.4 | 2.6E-3 | 6.6E-2 |
| GOTERM_BP_FAT | [autophagy](http://www.ebi.ac.uk/QuickGO/GTerm?id=GO:0006914) | 9 | 0.3 | 2.7E-3 | 6.6E-2 |
| GOTERM_BP_FAT | [mitochondrial electron transport, ubiquinol to cytochrome c](http://www.ebi.ac.uk/QuickGO/GTerm?id=GO:0006122) | 5 | 0.2 | 2.9E-3 | 7.0E-2 |
| GOTERM_CC_FAT | [late endosome](http://www.ebi.ac.uk/QuickGO/GTerm?id=GO:0005770) | 11 | 0.4 | 1.4E-2 | 7.3E-2 |
| GOTERM_CC_FAT | [Golgi-associated vesicle membrane](http://www.ebi.ac.uk/QuickGO/GTerm?id=GO:0030660) | 8 | 0.3 | 1.4E-2 | 7.5E-2 |
| SP_PIR_KEYWORDS | [cf(1)](http://www.uniprot.org/keywords/?query=cf(1)) | 5 | 0.2 | 8.9E-3 | 7.5E-2 |
| GOTERM_BP_FAT | [nuclear-transcribed mRNA catabolic process, nonsense-mediated decay](http://www.ebi.ac.uk/QuickGO/GTerm?id=GO:0000184) | 8 | 0.3 | 3.3E-3 | 7.7E-2 |
| GOTERM_BP_FAT | [negative regulation of cellular component organization](http://www.ebi.ac.uk/QuickGO/GTerm?id=GO:0051129) | 16 | 0.6 | 3.2E-3 | 7.7E-2 |
| GOTERM_BP_FAT | [respiratory electron transport chain](http://www.ebi.ac.uk/QuickGO/GTerm?id=GO:0022904) | 10 | 0.4 | 3.2E-3 | 7.7E-2 |
| GOTERM_BP_FAT | [monosaccharide metabolic process](http://www.ebi.ac.uk/QuickGO/GTerm?id=GO:0005996) | 33 | 1.2 | 3.4E-3 | 7.9E-2 |
| GOTERM_CC_FAT | [nuclear chromosome](http://www.ebi.ac.uk/QuickGO/GTerm?id=GO:0000228) | 17 | 0.6 | 1.5E-2 | 8.0E-2 |
| INTERPRO | [Thioredoxin-like](http://www.ebi.ac.uk/interpro/entry/IPR017936) | 15 | 0.5 | 5.0E-4 | 8.0E-2 |
| GOTERM_BP_FAT | [in utero embryonic development](http://www.ebi.ac.uk/QuickGO/GTerm?id=GO:0001701) | 28 | 1.0 | 3.5E-3 | 8.0E-2 |
| GOTERM_CC_FAT | [cell cortex](http://www.ebi.ac.uk/QuickGO/GTerm?id=GO:0005938) | 22 | 0.8 | 1.6E-2 | 8.2E-2 |
| INTERPRO | [Proteasome, alpha-subunit, conserved site](http://www.ebi.ac.uk/interpro/entry/IPR000426) | 7 | 0.3 | 4.4E-4 | 8.2E-2 |
| INTERPRO | [ATPase, AAA-type, conserved site](http://www.ebi.ac.uk/interpro/entry/IPR003960) | 10 | 0.4 | 4.8E-4 | 8.2E-2 |
| GOTERM_BP_FAT | [muscle organ development](http://www.ebi.ac.uk/QuickGO/GTerm?id=GO:0007517) | 25 | 0.9 | 3.6E-3 | 8.2E-2 |
| SMART | [WD40](http://smart.embl.de/smart/do_annotation.pl?DOMAIN=SM00320) | 41 | 1.5 | 3.2E-3 | 8.3E-2 |
| GOTERM_BP_FAT | [negative regulation of actin filament polymerization](http://www.ebi.ac.uk/QuickGO/GTerm?id=GO:0030837) | 7 | 0.3 | 3.8E-3 | 8.5E-2 |
| GOTERM_BP_FAT | [small GTPase mediated signal transduction](http://www.ebi.ac.uk/QuickGO/GTerm?id=GO:0007264) | 42 | 1.5 | 3.8E-3 | 8.6E-2 |
| SP_PIR_KEYWORDS | [wd repeat](http://www.uniprot.org/keywords/?query=wd%20repeat) | 41 | 1.5 | 1.1E-2 | 8.9E-2 |
| SP_PIR_KEYWORDS | [ubiquinone](http://www.uniprot.org/keywords/?query=ubiquinone) | 10 | 0.4 | 1.1E-2 | 9.0E-2 |
| SP_PIR_KEYWORDS | [thiol protease](http://www.uniprot.org/keywords/?query=thiol%20protease) | 18 | 0.6 | 1.2E-2 | 9.5E-2 |
| KEGG_PATHWAY | [Adherens junction](http://david.abcc.ncifcrf.gov/kegg.jsp?path=bta04520$Adherens%20junction&termId=470015067&source=kegg) | 21 | 0.8 | 1.1E-2 | 9.7E-2 |
| GOTERM_CC_FAT | [dynactin complex](http://www.ebi.ac.uk/QuickGO/GTerm?id=GO:0005869) | 4 | 0.1 | 1.9E-2 | 9.8E-2 |
| GOTERM_CC_FAT | [mitochondrial inner membrane presequence translocase complex](http://www.ebi.ac.uk/QuickGO/GTerm?id=GO:0005744) | 4 | 0.1 | 1.9E-2 | 9.8E-2 |

^1^ Number of differentially expressed genes (DEG) involved in the term

^2^ Percentage of DEG involved in the term (involved DEG/total DEG)
